# Interaction of Iron Oxide Nanoparticles with Macrophages Is Influenced Distinctly by “Self” and “Non-Self” Biological Identities

**DOI:** 10.1021/acsami.3c05555

**Published:** 2023-07-21

**Authors:** Yadileiny Portilla, Vladimir Mulens-Arias, Neus Daviu, Alberto Paradela, Sonia Pérez-Yagüe, Domingo F. Barber

**Affiliations:** † Department of Immunology and Oncology and Nanobiomedicine Initiative, Centro Nacional de Biotecnología (CNB-CSIC), Darwin 3, 28049 Madrid, Spain; ‡ Proteomics Facility, Centro Nacional de Biotecnología (CNB-CSIC), Darwin 3, 28049 Madrid, Spain

**Keywords:** magnetic nanoparticles, protein corona, mouse serum, human serum, macrophage activation

## Abstract

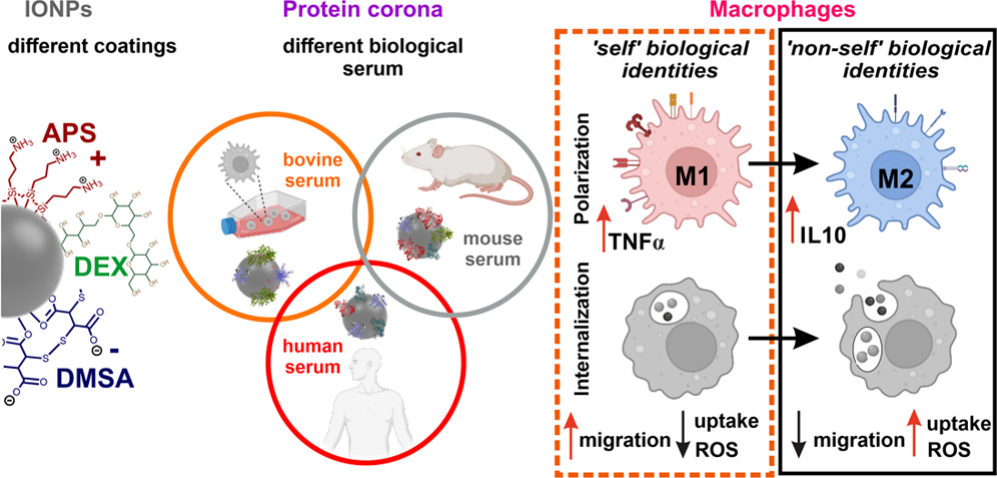

Upon contact with biological fluids like serum, a protein corona (PC) complex forms on iron oxide nanoparticles (IONPs) in physiological environments and the proteins it contains influence how IONPs act in biological systems. Although the biological identity of PC–IONP complexes has often been studied *in vitro* and *in vivo*, there have been inconsistent results due to the differences in the animal of origin, the type of biological fluid, and the physicochemical properties of the IONPs. Here, we identified differences in the PC composition when it was derived from the sera of three species (bovine, murine, or human) and deposited on IONPs with similar core diameters but with different coatings [dimercaptosuccinic acid (DMSA), dextran (DEX), or 3-aminopropyl triethoxysilane (APS)], and we assessed how these differences influenced their effects on macrophages. We performed a comparative proteomic analysis to identify common proteins from the three sera that adsorb to each IONP coating and the 10 most strongly represented proteins in PCs. We demonstrated that the PC composition is dependent on the origin of the serum rather than the nature of the coating. The PC composition critically affects the interaction of IONPs with macrophages in self- or non-self identity models, influencing the activation and polarization of macrophages. However, such effects were more consistent for DMSA-IONPs. As such, a self biological identity of IONPs promotes the activation and M2 polarization of murine macrophages, while a non-self biological identity favors M1 polarization, producing larger quantities of ROS. In a human context, we observed the opposite effect, whereby a self biological identity of DMSA-IONPs promotes a mixed M1/M2 polarization with an increase in ROS production. Conversely, a non-self biological identity of IONPs provides nanoparticles with a stealthy character as no clear effects on human macrophages were evident. Thus, the biological identity of IONPs profoundly affects their interaction with macrophages, ultimately defining their biological impact on the immune system.

## Introduction

Iron oxide nanoparticles (IONPs) are potent tools for clinical diagnosis and as drug delivery systems (DDS). This potential is not only steered by their intrinsic physicochemical properties, collectively called the physical identity (*e.g.*, magnetic susceptibility), but also by the modifications of their surface. Chemical modification of the IONP surface can endow nanoparticles (NPs) with different characteristics, influencing dispersion, stability, stealthiness, molecular specificity, anchoring points, and drug-loading capacity, facets that contribute significantly to the more widespread use of IONPs. Indeed, modifying the chemical surface of IONPs ultimately provides them with a synthetic identity that is defined by their surface charge, chemical bonds, and architecture. Both the physical and synthetic identities of IONPs can on the whole be controlled, and no external factors will temper with their main characteristics, which are intrinsically unique to the NP and its coating. However, a different picture is found when IONPs are dispersed in physiological fluids such as blood or the intracellular space, where an IONP’s colloidal status changes. Recently, there has been considerable interest in understanding the dynamic events taking place at the NP’s surface when in contact with these fluids, describing the qualitative and quantitative deposition of biomolecules, primarily proteins, that leads to the formation of the protein corona (PC). The resulting NP–PC complex is referred to as the biological identity of the NP, and it will dictate the majority of the biological interactions involving NPs in a physiological environment, such as their biodistribution, circulation time, cell internalization, immune responses, targeting, and drug delivery.^1−3^

It remains unclear whether or not the biological identity of NPs is dependent on their physicochemical properties, also known as synthetic properties. For instance, citrate- and riboflavin-coated IONPs appeared to showcase the divergence in the fetal bovine serum (FBS)-derived PC, as determined by a proteomic analysis, whereby riboflavin-coated IONPs associate with more Apolipoprotein E (ApoE), ApoA1, or albumin.^4^ Further modifying the IONP surface can affect the biological identity of the NPs. For instance, the attachment of a low pH insertion peptide and not the cycloRGD peptide to PEGylated IONPs enhanced IONP internalization by cells in the mononuclear phagocytic system when incubated in human serum (HS)-supplemented medium.^5^ However, we previously demonstrated that different coatings (dextran [DEX], 3-aminopropyl triethoxysilane [APS], or dimercaptosuccinic acid [DMSA]) did not result in different PC compositions after incubation in 10% FBS-supplemented medium, despite the distinct surface charges of the IONPs.^6^ Hence, different synthetic identities do not necessarily lead to different biological identities and thus, each NP system needs to be carefully studied to ascertain its physicochemical features and how these affect the NP’s biological identity, thereby enabling their therapeutic potential to be fully assessed.

Although enormous efforts have been made to better understand PC formation in relation to synthetic identity, most studies focused only on one physiological fluid at a time, neglecting the influence of the origin of the biological fluid on PC dynamics and composition. Most studies of the PC are carried out *in vitro*, primarily using FBS as it is a common *in vitro* cell culture supplement,^7,8^ although some addressed PC dynamics when generated in HS,^9,10^ mouse serum (MS),^10,11^ or alveolar fluid,^12^ or in media containing only one kind of protein.^13^ However, no transverse studies have been performed in which the same IONPs are maintained in sera of different origins with a view to understanding how the origin of the sera influences the biological identity of IONPs and their physiological behavior. Here, we define self biological identity as the system where both the PC-forming serum and the target cells are from the same species, as opposed to a non-self biological identity where the PC-forming serum and the target cells are from different species, the latter being the system most often employed when studying PC formation.

To our knowledge, little attention has been paid to date on the differences in PC composition provoked by sera of different origins and the biological impact of these, particularly in relation to IONPs. However, in one attempt to understand this process, magnetic mesoporous silica NPs were preincubated with PBS supplemented with either 10% HS or FBS to deposit a PC. Curiously, the formation of an HS-derived PC on the magnetic mesoporous NPs better overcame the cytotoxicity of pristine magnetic mesoporous NPs to HepG2 cells than an FBS-derived PC, suggesting some kind of ‘self-recognition’.^14^ Silica NPs with different surface charges were also exposed to medium supplemented with FBS or HS, which led to PCs with distinct protein compositions or synthetic identity (surface charge).^15^ More recently, gold and silica NPs were exposed to medium supplemented with HS, FBS, or MS, and their PCs were analyzed by nanoflow liquid chromatography–electrospray ionization-tandem mass spectroscopy.^16^ As a result, interspecies differences were found in PC composition, whereby the HS-derived PC differed substantially from those derived from FBS or MS in terms of their biological classification, although the biological consequences of these differences were not assessed.^17^ Nonetheless, the impact of the origin of the serum on IONP PC formation has yet to be addressed, even though they have become a widely used nanosystem.

In light of the above, we sought to comprehensively analyze the biological identity of 12.0 (±1.2 nm) diameter IONPs with three different synthetic identities: positively charged APS-IONPs; neutrally charged DEX-IONPs; and negatively charged DMSA-IONPs. Notably, these IONPs not only exhibit differences in terms of their surface charge but also, in their chemical bonds (*e.g.*, −SH, −OH, −SiO−) and architecture. Thus, we selected these three IONPs as representative of surface charge and chemical bonds, and based on their theranostic potential, and we considered two models of biological identity: self biological identity (human or mouse) or non-self biological identity (bovine and human or mouse and bovine). In this way, we cover a large range of the *in vitro* and *in vivo* experimental scenarios currently used to test IONPs, and the self biological identity model in particular provides valuable insights into *in vivo* approaches.

It is broadly accepted that nanomaterials like IONPs primarily interact with cells of the mononuclear phagocytic system in reticular connective tissues. As part of the innate immune system, IONP-challenged macrophages will initiate a pleid of biological processes, leading to either activation^18,19^ or immunosuppression.^20^ Many of the effects of IONPs on macrophages are associated with the production of reactive oxygen species (ROS) due to iron cations, leading to exacerbated oxidative stress.^19,21^ In addition, IONPs seem to promote macrophage polarization through the engagement of toll-like receptors (TLRs), mostly TLR4, mediated by molecular domains present in the IONP coating.^22,23^ Therefore, the effects of IONPs on macrophages appear to be mainly linked to their physical and surface identities. However, it is unclear how the biological identity, including selfness, fine-tunes IONP-induced macrophage polarization. Thus, we targeted macrophages *in vitro* to elucidate the impact of the PC “selfness” on macrophage polarization. As such, IONPs were cultured in serum-supplemented medium and their colloidal status was assessed after 24 h, evaluating their size, aggregation, and surface charge.

A label-free proteomic analysis was also used to elucidate the PC composition of each IONP when maintained in the presence of serum from different origins (human, bovine, or murine). The differences in PC composition are more closely related to the biological serum than the coatings, indicating that the biological identity of IONPs is highly dependent on their physiological environment. Indeed, different biological identities activated and polarized macrophages distinctly, as seen most clearly for DMSA-IONPs. Indeed, the self biological identity of DMSA-IONPs activates and elicits M2 polarization of murine macrophages, whereas the non-self biological identity favors M1 polarization, as demonstrated by their cytokine expression and ROS production. The opposite effect was observed in the human model, whereby a self biological identity of DMSA-IONPs promotes mixed M1/M2 polarization with an increase in ROS production. However, a non-self biological identity of DMSA-IONPs makes the NPs stealthy, as no effect on human macrophages was detected. Together, we demonstrate that the biological identity of IONPs significantly influences their interaction with macrophages and ultimately, it defines their impact on the immune system. These results provide insights into how IONP-induced macrophage polarization can be modulated through the biological identity of IONPs.

## Results and Discussion

### Synthesis and Characterization of the IONPs

IONPs are currently among the most intensely studied metallic NPs given their potential theranostic uses in biomedical fields like cancer^24^ or as DDS.^25^ Furthermore, it is now accepted that the biomolecular PC that forms dynamically on NPs defines their biological identity and ultimately, influences their fate. However, it is unclear whether sera of diverse origin influence the dynamic features and composition of the PC formed. Therefore, we investigated the interaction of IONPs with sera of different biological sources, first synthesizing the uncoated IONPs by co-precipitation,^26^ producing spherical particles ∼12 nm in diameter (Figure S1). We chose IONPs of equal iron oxide core to rule out the influence of this parameter in our comparison. We did not choose the hydrodynamic diameter as it is influenced by the coating layer and the medium in which the nanoparticles diffuse and, thus, difficult to control. The IONPs were coated with one of three different layers (APS, DEX, or DMSA), providing them with distinct surface charges and chemical properties. Although we have used these types of IONPs previously,^6^ here we present the physicochemical characteristics of the batches prepared for these specific experiments as they may differ slightly from the data obtained from previous batches. The IONPs had a negative (−24.8 ± 4.9 mV, DMSA), neutral (−1.7 ± 0.1 mV, DEX), or positive (+25.8 ± 3.8 mV, APS) surface charge (Table 1), and they adopted a well-dispersed colloidal state with a hydrodynamic diameter of 64.7 ± 12.2, 116.4 ± 10.1, and 112.3 ± 5.8 nm, respectively (Table 1) in water.

**Table 1:** Physicochemical Characteristics of APS-, DEX-, and DMSA-IONPs in Water

physicochemical characteristics	APS-IONPs	DEX-IONPs	DMSA-IONPs
size of iron oxide core (nm)	12.0 ± 1.2	12.0 ± 1.2	12.0 ± 1.2
hydrodynamic size (nm)	112.3 ± 5.8	116.4 ± 10.1	64.7 ± 12.2
polydispersity Index (PDI)	0.237	0.148	0.131
ζ-potential (mV)	+25.8 (±3.8)	–1.7 (±0.1)	–24.8 (±4.9)

Having synthesized the IONPs with different superficial charges, bonds, and architectures, we proceeded to study the dynamics and composition of the PC deposited onto the NPs when in contact with sera from different biological sources.

### Analysis of Dynamic PC Formation Based on IONP Coating, the Culture Medium, and the Serum Origin

**Figure 1:**
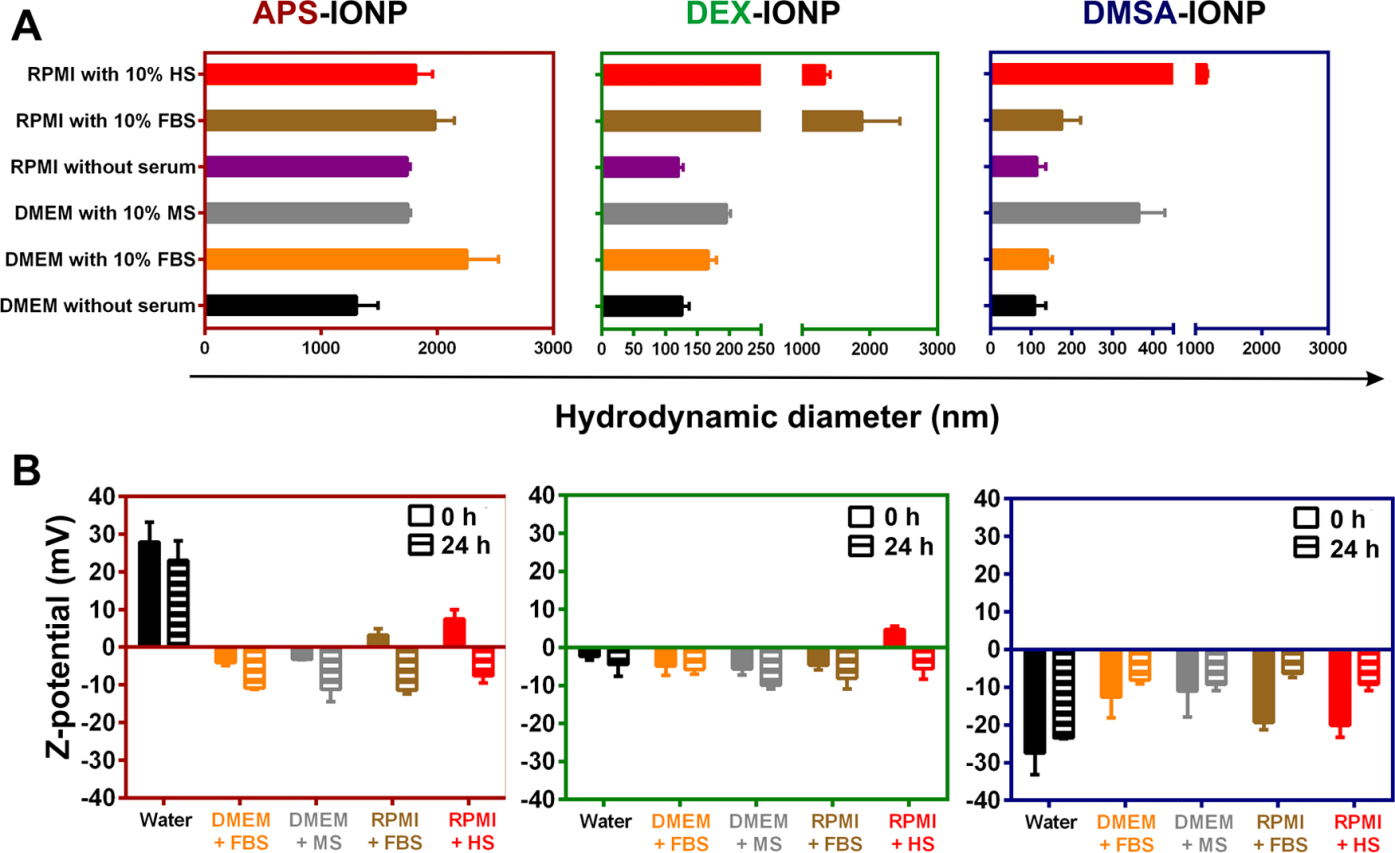
Protein corona deposition on APS-, DEX-, and DMSA-IONPs depends on the type of serum. (A) Hydrodynamic size and (B) the *Z*-potential before and 24 h after incubation in 10% serum-supplemented DMEM or RPMI, as determined by dynamic light scattering (DLS). In (B), the filled bars represent the charge of the IONPs at 0 h and the stippled bars represent the surface charge of the IONPs after 24 h in the medium indicated. Three replicates were monitored throughout the experiments.

The biological identity of NPs can be defined as the shift in NP identity due to protein (and other biomolecule) deposition onto the NP surface, altering the way NPs interact with cells.^2^ Thus, an in-depth analysis of the PC formed on the IONPs is crucial to comprehend the behavior of NPs in living systems. We first followed PC formation on each IONP by measuring the hydrodynamic diameter and *Z*-potential over time, which can be taken as a surrogate for the dynamic deposition of serum-stemmed proteins on the surface (Figure 1). Since previous studies revealed that the PC thickness stabilizes after an ∼24 h incubation in serum-supplemented medium, regardless of the serum origin (Figure S2),^6^ we monitored the hydrodynamic size and Z-potential of the IONPs maintained in serum for 24 h. Importantly, two different culture media were used, Dulbecco Modified Eagle’s medium (DMEM) and Roswell Park Memorial Institute 1640 medium (RPMI), as these culture media affect the dynamic deposition of the PC distinctly.^27^ In addition, three different sera commonly used in cell culture were assayed, rendering the following incubation media: (1) 10% FBS-supplemented DMEM; (2) 10% MS-supplemented DMEM; (3) 10% FBS-supplemented RPMI; and (4) 10% HS-supplemented RPMI. As controls, we incubated IONPs (125 μg Fe/mL) in media without any supplement (DMEM or RPMI), thereby covering a wide range of the standard media commonly used for *in vitro* cell culture.

The APS-IONPs increased their hydrodynamic diameter 1.7-fold after 24 h in FBS-supplemented DMEM and 1.3-fold in MS-supplemented DMEM relative to the basal medium (Figure 1A). By contrast, the hydrodynamic diameter of APS-IONPs increased 1.2-fold in FBS-supplemented RPMI and it remained unchanged in HS-supplemented RPMI relative to the APS-IONPs maintained in basal RPMI (Figure 1A). There was a 1.3-fold increase in the hydrodynamic diameter of the neutral DEX-IONPs, in FBS-supplemented DMEM and an increase of 1.6-fold in MS-supplemented DMEM relative to the basal DMEM (Figure 1A). Notably, we observed a 15.6-fold increase in the hydrodynamic diameter of DEX-IONPs when they were incubated in FBS-supplemented RPMI and an 11.1-fold increase in HS-supplemented RPMI relative to the basal medium (Figure 1A). Therefore, while APS- and DEX-IONPs behave similarly in serum-supplemented DMEM, a much thicker PC appears to be deposited on the neutral DEX-IONPs in RPMI medium regardless of the serum than on the positively charged APS-IONPs. Nonetheless, the large increase in the hydrodynamic diameter of DEX-IONPs in the different conditions might also reflect an increment of the aggregation state of the nanoparticles. Finally, DMSA-IONPs increased their hydrodynamic diameter 1.3-fold in FBS-supplemented DMEM and 3.4-fold when it was supplemented with MS (Figure 1A). While DMSA-IONPs showed hardly any increase in their hydrodynamic diameter in FBS-supplemented RPMI (1.5-fold), much larger protein-conjugates (10.2-fold) were detected in HS-supplemented RPMI (Figure 1A). We noticed that while there was not much difference in the hydrodynamic diameter of APS- and DMSA-IONPs in DMEM/RPMI, the increase in diameter of DEX-IONPs in FBS-supplemented RPMI was more than 10-fold that in FBS-supplemented DMEM, suggesting that RPMI favors protein deposition on neutral NPs. Indeed, there was a consistently larger increase in the hydrodynamic diameter of DEX-IONPs in serum-supplemented RPMI, which might also be related to the formation of large aggregates given the ionic strength of the medium and the nature of the PC formed, as we have analyzed previously^6^ (Figure S3). Together, these data indicated that the PC of IONPs had a distinct thickness depending on the NP’s surface charge, the cell culture medium, and the origin of the animal serum.

The three IONPs exhibited a negative *Z*-potential when incubated in serum-supplemented media, as a result of the PC deposition and irrespective of the surface charge in water (Figure 1B). Indeed, the positively charged APS-IONPs acquired a negative charge as the PC formed, while the neutral DEX-IONPs became negatively charged and the negatively charged DMSA-IONPs remained negative, although to a lesser extent.

### Proteomic Profiling of the PC Formed in Sera of Different Biological Origin

While the sera of different animal origins produce different PCs, at least in terms of thickness, it remains unclear if their protein composition also changes. Therefore, we used a label-free proteomic approach to analyze the PC deposited on the IONPs after 24 h in serum-supplemented media, recovering the IONPs magnetically and resuspending them in protein extraction buffer. The protein extracts were separated by sodium dodecyl sulfate polyacrylamide gel electrophoresis (SDS-PAGE), trypsin-digested, and the resulting peptides were finally identified and analyzed using a spectral counting proteomic technique. The PC thickness did not differ greatly in serum-supplemented DMEM (1.3- to 3.4-fold larger), while the considerable increase in the hydrodynamic diameter of DEX- and DMSA-IONPs in serum-supplemented RPMI (10.2- to 15.6-fold) could be related to the formation of NP aggregates. As such, we chose DMEM to interrogate the PC protein composition. Significantly, the sera were not inactivated or decomplemented in order to reproduce the complete protein profile in a physiological scenario.

**Figure 2:**
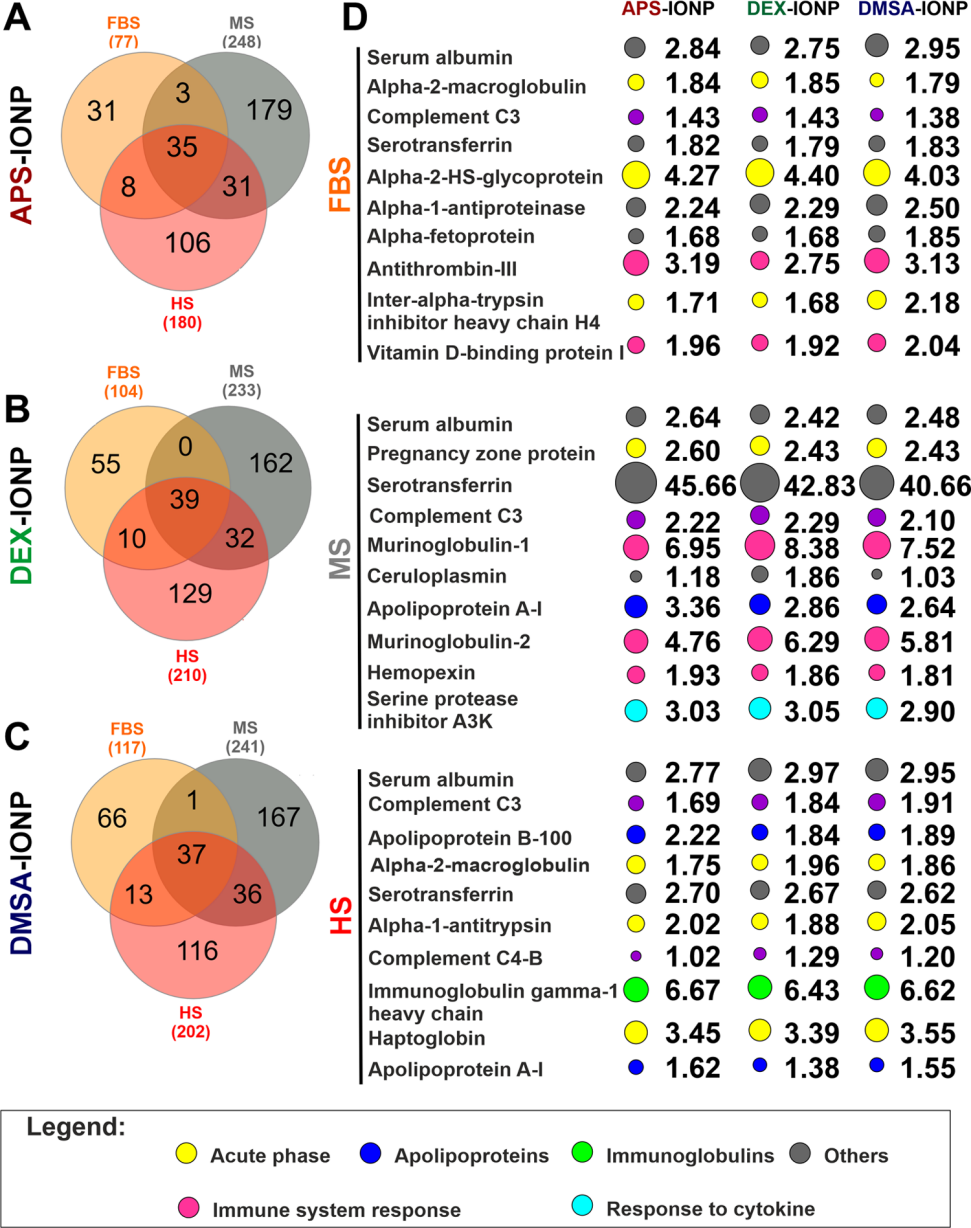
Proteomic characterization of the PC associated with IONPs depending on the serum type and coating of the IONPs. (A–C) Venn diagrams showing the number of bovine (in orange), mouse (in gray), and human proteins in the coronas (in red) on the surface of APS-, DEX-, and DMSA-IONPs. (D) Heat map of the top 10 proteins formed from APS-, DEX-, and DMSA-IONPs depending on the type of biological serum (top, FBS; middle, MS; and bottom, HS).

According to the Venn diagrams, MS contributed the largest number of proteins to the PC deposited on the IONPs regardless of their coating, more than FBS or HS. For example, we detected 31 (40.3%) unique FBS-derived proteins, 179 (72.2%) unique MS-derived proteins, and 106 (58.9%) unique HS-derived proteins on APS-IONPs. In addition, 35 (14.1%) proteins were common to FBS, MS, and HS on APS-IONPs (Figure 2A). Likewise, we detected 55 (52.9%) unique proteins in the FBS-derived PC on DEX-IONPs, 162 (69.5%) in that generated in MS, and 129 (61.4%) in the presence of HS, with 39 (16.7%) proteins common to all of the sera (Figure 2B). Finally, the PC of DMSA-IONPs had 66 (56.4%), 167 (69.3%), and 116 (57.4%) unique FBS-, MS-, and HS-derived proteins, as well as 37 (15.4%) proteins common to all of the sera (Figure 2C).

A more in-depth functional analysis revealed 8.9% of proteins in the HS-derived APS-IONP PC had molecular regulator activity, as well as 27.8% with catalytic activity, 21.5% with binding capacity, 0.8% with molecular transducer activity, 1.3% with transporter activity, 1.3% with ATP-dependent activity and 1.2% with transcription regulatory activity (Figure S4A). By contrast, the FBS- and MS-derived PCs of APS-IONPs only contained proteins with binding (FBS-derived, 21.4%; MS-derived, 27.1%), catalytic (FBS-derived, 24.8%; MS-derived, 22%), and molecular regulator activities (FBS-derived, 0.8%; MS-derived, 1.7%: Figure S4A). We did not detect differences among the serum-derived PC deposited on DEX-IONPs in terms of functional classification, in which proteins with binding (27.1% from FBS; 25.0% from MS; 19.3% from HS) or catalytic activity (27.1% from FBS; 23.2% from MS; 24.1% from HS) prevail, with more molecular regulators (8.3% from FBS; 10.7% from MS; 10.8% from HS: Figure S4A). The DMSA-IONP PC derived from FBS, MS, and HS had proteins associated with the categories of binding (22.4% from FBS, 21.7% from MS, and 20% from HS) or catalytic activity (23.4% from FBS, 23.9% from MS and 25.6% from HS), and the proportion of molecular regulators was similar between the sera (8.4% from FBS, 10.9% from MS and 8.9% from HS). However, the HS-derived PC associated with DMSA-IONPs had three additional functional categories that were not identified in the FBS- and MS-derived PC (Figure S4A).

Nonetheless, the most remarkable differences in the serum-derived IONP PCs were evident in the classification of the proteins in terms of their physiological function (apolipoproteins, proteins of the complement system, and immunoglobulins) with the aid of the Panther Gene List software that recognizes Gene Ontology annotations and associates them to the proteins identified. As such, immunoglobulins were enriched in the PCs derived from the MS and HS, regardless of the IONP coating (Figure S4B), whereas MS- and HS-derived proteins of the complement system and apolipoproteins were less enriched in the IONP-associated PC (Figure S4B). Such immunoglobulin enrichment in the MS- and HS-derived PC might reflect the relative abundance of these molecules in the whole serum. Indeed, immunoglobulins are among the most abundant proteins in the PC deposited on NPs incubated in full HS, such as SiO_2_ NPs;^28^ COOH-, NH_2_-, and −CH_3_-surface-modified SiO_2_ NPs;^29^ gold NPs;^30,31^ and polystyrene (PS) NPs.^32^ The enrichment of immunoglobulins in MS- and HS-derived PCs may facilitate and modulate their uptake by cells through Fc receptors and cell activation.^33^ However, it has also been demonstrated that immunoglobulins may undergo aggregation and denaturation during their dynamic deposition as part of the PC, potentially losing some of their biological activity.^34^ If the structure of immunoglobulins is preserved when they are adsorbed onto NPs, they can still target the IONPs to Fcγ receptor-expressing cells. Furthermore, immunoglobulin adsorption can facilitate the opsonization of NPs once they are in contact with plasma by promoting the adsorption of C3 complement protein.^35^ The immunoglobulins in the PC can modulate the adhesion of NPs to activated endothelial cells,^36^ with both IgA and IgM limiting the adhesion of poly(lactic-*co*-glycolic) acid (PLGA) carriers to activated endothelial cells in human blood. Therefore, the biological identity of IONPs can be affected significantly by the presence of immunoglobulins.

By contrast, apolipoprotein and complement-related proteins are the only proteins enriched in the FBS-derived PCs regardless of the IONP coating. The lack of immunoglobulins in all FBS-stemmed PCs might be related to the relatively low level of immunoglobulins in FBS compared to newborn and adult calf sera.^37^ While IgM is apparently present to some extent at fetal stages, it is not evident post-partum when mammals start to produce substantially more immunoglobulin upon class-switching, which leads to predominant IgG and IgA production.^38^ Thus, immunoglobulins are more frequent in newborn and adult mammals.

The abundance of apolipoproteins in FBS-stemmed PCs might endow NPs with immunomodulatory features. Apolipoproteins are central to the transport and metabolism of lipids and cholesterol in the bloodstream and across biological barriers.^39^ Notably, apolipoproteins also exert enzyme co-factor activity and they act as cell surface receptor ligands *per se*, such as for the low-density lipoprotein receptor (LDLR), the LDLR-related protein (LRP), the very low-density lipoprotein (VLDL), the apolipoprotein E receptor 2 (ApoER2), and heparan sulfate proteoglycan (HSPG).^40,41^ More importantly, apolipoproteins are well known to interact with the immune system by complexing immunomodulatory lipids (*e.g.*, S1P and lysophosphatidylcholines), and associating with immune regulatory proteins like those found as part of the high-density lipoprotein (HDL) particles.^42^ Various studies have demonstrated that nanomaterial-protein complexes can mimic lipoprotein particles, facilitating cell uptake by the brain’s capillary endothelial cells via LDLR-mediated endocytosis.^43,44^ In addition, the complement system plays a crucial role in the innate immune system by recognizing foreign entities, which leads to the formation of a membrane attack complex (MAC) that detects and removes harmful pathogens.^45^ Together, the differences in immunoglobulin, complement, and apolipoprotein families might dictate the properties of the PC-coated IONPs and their interactions with the innate immune system.

These results differ from previous studies where apolipoproteins were the most frequent protein in the PC formed when negatively charged gold and SiO_2_ NPs were incubated in sera of different origins (HS, BS, or MS). By contrast, the three IONPs studied here promoted the deposition of apolipoproteins from HS and MS, constituting >60 and >85% of the total proteins, respectively, whereas the FBS-derived PC exhibited no detectable apolipoproteins. Indeed, while MS contributed the highest proportion of apolipoproteins to the PCs deposited onto negative gold and SiO_2_ NPs, MS was also the serum that contributed the most apolipoprotein to the IONP PCs here (>85%), regardless of the coating.^17^ Indeed, as indicated previously, PC formation appears to be more dependent on the serum origin than the IONP composition. Therefore, the physiological environment in which IONPs exert their activity is likely to profoundly affect their biological identity. More detailed profiling revealed that the α-2-HS-glycoprotein, also known as fetuin-A (FetA: 4.27-fold, 4.40-fold, and 4.03-fold enrichment), antithrombin-III (ATIII: 3.19-fold, 2.75-fold, 3.13-fold enrichment) and serum albumin (2.84-fold, 2.75-fold, 2.95-fold enrichment) were the three most abundant proteins on the PC derived from FBS and deposited on APS-, DEX-, and DMSA-IONPs, respectively (Figure 2D, top). Notably, the abundance of both α-2-HS-glycoprotein (AHSG) and serum albumin in the FBS-derived PC might reflect the relative abundance of these proteins in FBS, as demonstrated previously,^7^ where they represent 26.26 and 35.69% of all proteins, respectively.

The relative abundance of ATIII in FBS-derived PCs is more curious, as it is a protein that only represents 0.33% of the total protein in FBS. Of the 10 overrepresented proteins in the FBS-derived PCs, AHSG,^46,47^ α-2-macroglobulin (α2M),^48^ and the vitamin D-binding protein^49^ have the potential to directly activate the innate immune system, particularly acting on macrophages and indicating their capacity to modulate the immune system.

The MS-derived PC portrays different enriched proteins, with serotransferrin (45.66-fold, 42.83-fold, and 40.66-fold enrichment), murinoglobulin-1 (Mug1: 6.95-fold, 8.38-fold, and 7.52-fold enrichment) and murinoglobulin-2 (Mug2: 4.76-fold, 6.29-fold, and 5.81-fold enrichment) the most abundant proteins in the APS-, DEX-, and DMSA-IONP PCs, respectively (Figure 2D, middle). Some proteins in the MS-derived PC can also modulate macrophage activation, such as Mug1/2 that can regulate the migratory behavior of macrophages through their protease inhibitor activity and that of other cells in the innate immune system.^50^ Similarly, the serine protease inhibitor A3K has been linked to a protective role in some pro-inflammatory scenarios.^51^ Other proteins enriched in the MS-derived PCs exert anti-inflammatory activity, such as ApoA1^52^ and the heme-scavenger hemopexin (HPX),^53^ suggesting that MS-derived PCs might exert global anti-inflammatory effect.

Finally, the immunoglobulin γ-1 heavy chain (6.67-fold, 6.43-fold, and 6.62-fold enrichment), the haptoglobin (3.45-fold, 3.39-fold, and 3.55-fold enrichment), and serum albumin (2.77-fold, 2.97-fold, and 2.95-fold enrichment) are the three most abundant proteins in the HS-derived PC deposited on APS-, DEX-, and DMSA-IONPs, respectively (Figure 2D, bottom). When looking specifically at the top overrepresented proteins in the HS-derived PCs, some have proven anti-inflammatory activity like haptoglobin, which in concert with hemoglobin triggers CD163-mediated macrophage activation toward an M2 phenotype.^54^ Likewise, α-1-antitrypsin (AAT) promotes an anti-inflammatory environment, antagonizing some autoimmune diseases,^55,56^ while α2M can instigate macrophage activation toward a pro-inflammatory phenotype.^48,57^ Therefore, global immune regulation of HS-derived PCs might reflect the balance of all of these putative effects.

The relative abundance of the proteins in HS-derived PCs described here differs from those defined elsewhere, such as the PC deposited on PS and gold NPs. When aminated, carboxylated, or bare, the most abundant proteins on PS NPs incubated with full HS were histidine-rich glycoproteins (>8%), ApoA-I (>3.7%), and plasminogen (>2.8%), irrespective of surface charge.^28^ However, when citrate gold NPs were incubated with full HS, complement family members C3, C4-B, and C4-A were the most abundant proteins.^58^ Such divergent protein compositions can be explained by the different surface chemical bonds, architecture, and core chemistry of the NPs.

Noteworthy, we have analyzed the PC formation onto IONP incubated for 24 h *in vitro* as a manner to model the process. However, since PC formation is a dynamic process,^59,60^ it is influenced by not only the time but also the microenvironment in which the IONPs pass through. That is the case for the intracellular transit of nanoparticles, which promotes a dynamic PC in which a protein exchange occurs when nanoparticles pass from blood to lysosome, and from the lysosome to the cytoplasm. This intracellular journey provokes an exchange of different chaperons and metabolic proteins that eventually affect the level of autophagy.^61^ Besides, the microenvironment fluids such as the bronchoalveolar lavage fluids can determine the way lipid-based nanoparticles interact with lung epithelial cells and lung resident macrophages.^62^ Parameters such as protein composition, protein concentration, ionic strength, and pH can drive different PC kinetic and composition that dynamically change over time. Therefore, it is expected that PC deposited onto IONPs be different when in contact with diverse biological fluids such as bloodstream, cerebrospinal fluid, interstitial fluids, and intratumoral fluids. Future experiments should address how specific microenvironment-driven PC deposited onto IONP affect their interaction with monocytes/macrophages.

### Influence of “Self” and “Non-Self” Biological Identity on Macrophage IONP Uptake

As demonstrated previously, PC formation can alter the surface charge of NPs due to the collective charge of the proteins deposited, also endowing NPs with their so-called biological identity that ultimately controls the physiological response they provoke. Cell membranes exhibiting a negative charge might repel PC-coated IONPs, hindering their entry into the cell. However, as the PC often contains protein cognates for cell membrane receptors (*e.g.*, proteins of the complement system and immunoglobulins: Figure 2D), NPs can overcome electrostatic barriers and enhance their cell uptake through receptor-mediated endocytosis. Indeed, the uptake of positively charged gold NPs of similar size and charge by murine macrophage-like RAW264.7 cells positively correlates with enrichment in the complement protein C4BPA, while there is a negative correlation with the presence of the immunoglobulin IGLC2.^63^ Similar effects of PC protein composition on NP uptake were observed by incubating PS NPs in heat-inactivated or non-heat-inactivated fetal calf serum, with heat-inactivation substantially diminishing NP uptake by A549 cells.^64^ As mentioned, apolipoproteins also play an essential role in the innate system and, indeed, ApoB-containing lipoprotein uptake by macrophages underlies the formation of atherosclerosis plaques that provokes an inflammatory response.^65^ Thus, the PC protein composition can dramatically alter NP cell uptake due to the incorporation of cognates and their interaction with their corresponding cell membrane receptors.

We sought to elucidate whether a self and or non-self biological identity affects IONP internalization by macrophages. Thus, we set up two models: (1) a mouse model, in which the murine cell line RAW264.7 was incubated with IONPs in FBS (non-self identity) or MS (self identity); (2) a human model, in which human THP1 cells were incubated with IONPs in FBS (non-self identity) or 10% HS (self identity). Note that both models were based on FBS as the standard *in vitro* cell culture system and the species (murine or human) corresponding to an environment closer to the *in vivo* systems. We did not detect cell toxicity across the range of IONP concentrations tested (up to 250 μg/mL: Figure S5). In the murine model, RAW264.7 cells took up more APS-IONPs (69.1 ± 15.9 pg/cell) than DMSA-IONPs (39.0 ± 5.4 pg/cell) and DEX-IONPs (16.0 ± 5.5 pg/cell) when incubated in FBS (Figure S6A). Similarly, the RAW264.7 cells internalized more APS-IONPs (110.3 ± 4.5 pg/cell) when incubated in MS, while the internalization of DEX-IONPs (42.0 ± 12.3 pg/cell) and DMSA-IONPs (61.9 ± 8.8 pg/cell) was similar when cells were incubated in MS (Figure S6A). Comparing the two sera, both APS- and DEX-IONPs were internalized at a significantly higher rate in MS, superior to DEX-IONPs, suggesting that an MS-derived PC might potentiate NP internalization. In the human model, the APS-IONPs were also those best internalized by THP1 cells in both FBS (73.4 ± 3.3 pg/cell) and HS (204.5 ± 56.2 pg/cell), while there were no significant differences in the cell internalization of the DEX-IONPs (FBS 67.9 ± 9.1 pg/cell or HS 22.5 ± 12.1 pg/cell) and DMSA-IONPs (FBS 2.4 ±1.0 pg/cell or HS 1.7 ± 1.0 pg/cell), regardless of the serum (Figure S6B). The APS-IONPs exhibited a better internalization rate when incubated in HS, indicating that human self identity of IONPs might also potentiate APS-IONP internalization. Together, it appears that DMSA-IONPs are internalized by the same cells at a similar rate regardless of their biological identity, while the APS-IONPs exhibit a higher internalization rate by cells in a self identity system. This latter scenario was also true for DEX-IONPs in the murine model (Figure S7).

**Figure 3:**
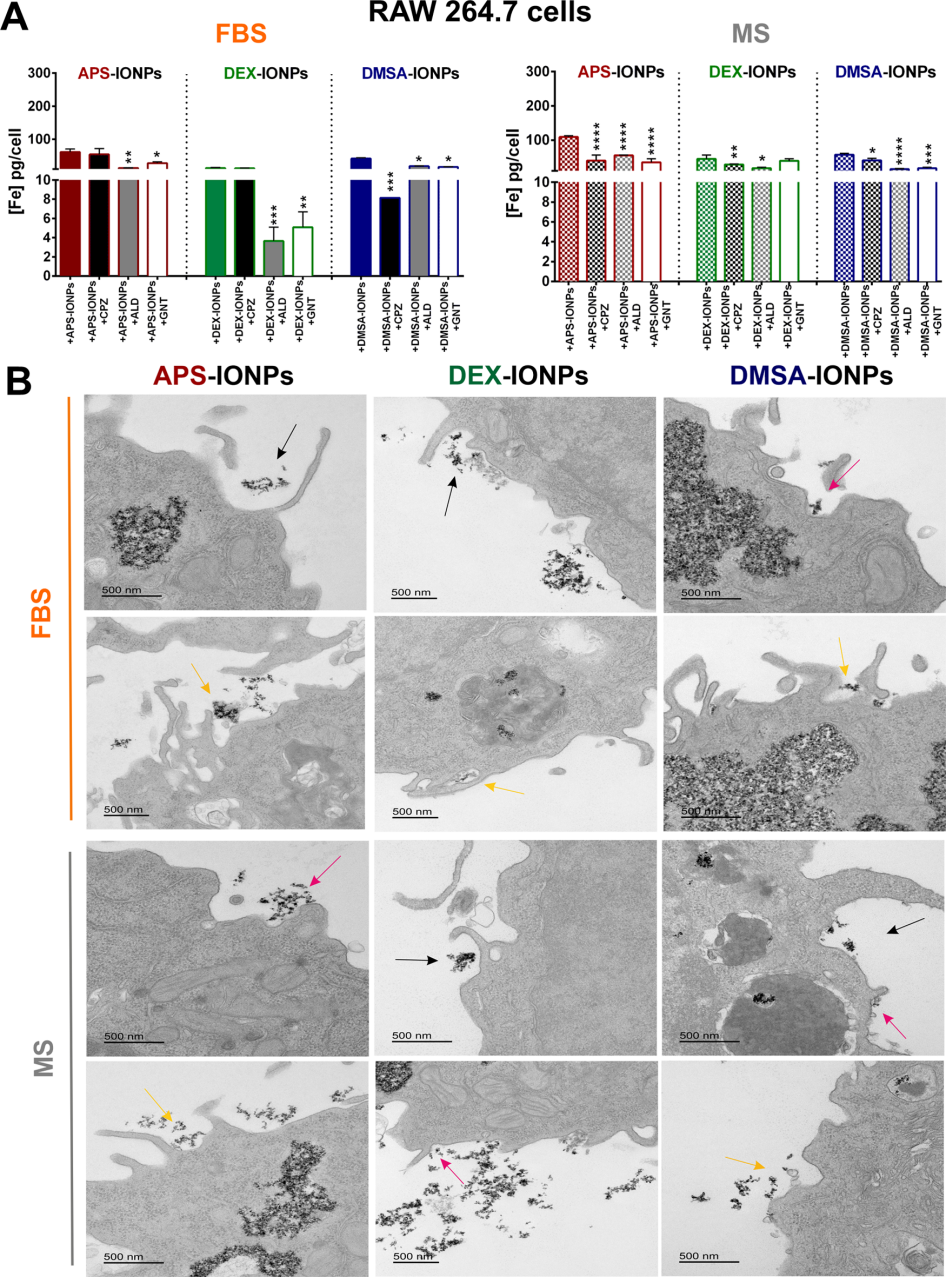
Internalization of IONPs with different biological identities by murine RAW264.7 cells. (A) Endocytic pathways used by APS-, DEX-, and DMSA-IONPs assayed in RAW264.7 cells incubated in medium with FBS (left) and MS (right). We used specific chemical inhibitors to block some of the main pathways of internalization: chlorpromazine (CPZ), an inhibitor of clathrin-mediated endocytosis; amiloride (AML), an inhibitor of macropinocytosis; and genistein (GNT) to inhibit caveolae-dependent endocytosis. (B) Transmission electron microscopy (TEM) analysis of the typical membrane structures associated with IONP internalization indicated by colored arrows: clathrin-mediated endocytosis (pink arrows clathrin-coated pits); caveolae-mediated endocytosis (yellow arrows flask-shaped structures); macropinocytosis (black arrows macropinosomes), in this case RAW264.7 cells were treated with IONPs but in the absence of endocytosis inhibitors. Scale bar: 500 nm. The data (mean ± standard deviation (SD)) are representative of three independent experiments and analyzed with a two-way analysis of variance (ANOVA) and a Tukey’s multiple comparisons test: *p < 0.05, **p < 0.01, and ***p < 0.001.

Having established the internalization rate for each IONP depending on their biological identity, we further analyzed the endocytotic mechanism in the same cell models (murine and human) using specific chemical inhibitors to block the main internalization pathways: chlorpromazine to inhibit clathrin-mediated endocytosis; amiloride to inhibit macropinocytosis; and genistein to inhibit caveolae-dependent endocytosis. In the murine model (RAW264.7 cells), the macrophage-like cells were cultured for 24 h in DMEM supplemented with FBS or MS and then treated with each inhibitor for 2 h prior to exposing them for another 24 h to APS-, DEX-, or DMSA-IONPs (125 μg Fe/mL). When RAW264.7 cells were exposed to amiloride (13.5 ± 0.2 pg/cell) or genistein (28.1 ± 4.2 pg/cell) but not chlorpromazine (54.4 ± 7.4 pg/cell), and then incubated in FBS, there was a significant reduction in iron content relative to untreated cells (61.5 ± 9.0 pg/cell), indicating the involvement of the caveolae-mediated endocytosis and macropinocytosis (Figure 3A,B).

However, when incubated in MS, pretreatment with chlorpromazine (40.2 ± 5.8 pg/cell), amiloride (55.3 ± 1.0 pg/cell) or genistein (35.0 ± 10.8 pg/cell) reduced iron content equally relative to untreated cells (109.0 ± 3.9 pg/cell), indicating that the MS-derived PC facilitates APS-IONP internalization via clathrin (Figure 3A,B). A strong involvement of caveolae-dependent endocytosis and macropinocytosis was observed for the DEX-IONPs incubated in FBS since amiloride (3.7 ± 1.4 pg/cell) and genistein (5.1 ±1.6 pg/cell) significantly reduced IONP internalization relative to the untreated cells (13.1 ± 1.8 pg/cell: Figure 3A). Although chlorpromazine appeared to produce a slight reduction in internalization (28.4 ± 1.8 pg/cell) by RAW264.7 cells when incubated with DEX-IONPs in MS, as did amiloride (17.9 ± 2.8 pg/cell) relative to untreated cells (44.9 ± 11.2 pg/cell), the reduction was almost negligible compared to the effects in FBS (Figure 3A,B). Therefore, the internalization of FBS-derived PC-coated DEX-IONPs is more dependent on macropinocytosis and caveolae-dependent endocytosis than MS-derived PC-coated DEX-IONPs. Although RAW264.7 cells endocytosed less iron when pretreated with all of the inhibitors of endocytosis assayed here, chlorpromazine (8.2 ± 1.0 pg/cell) diminished cell iron content more than amiloride (18.8 ± 1.1 pg/cell) and genistein (16.5 ± 1.0 pg/cell) in FBS, indicating the preferential involvement of clathrin-dependent endocytosis in DMSA-IONP internalization (Figure 3A,B). However, incubation in MS partially rescued iron internalization after pretreatment with the chlorpromazine (40.8 ± 6.1 pg/cell), although it was still significantly lower than that in untreated cells (57.4 ± 4.1 pg/cell), indicating that the MS-derived PC drives DMSA-IONPs toward an endocytotic pathway (Figure 3A,B).

**Figure 4:**
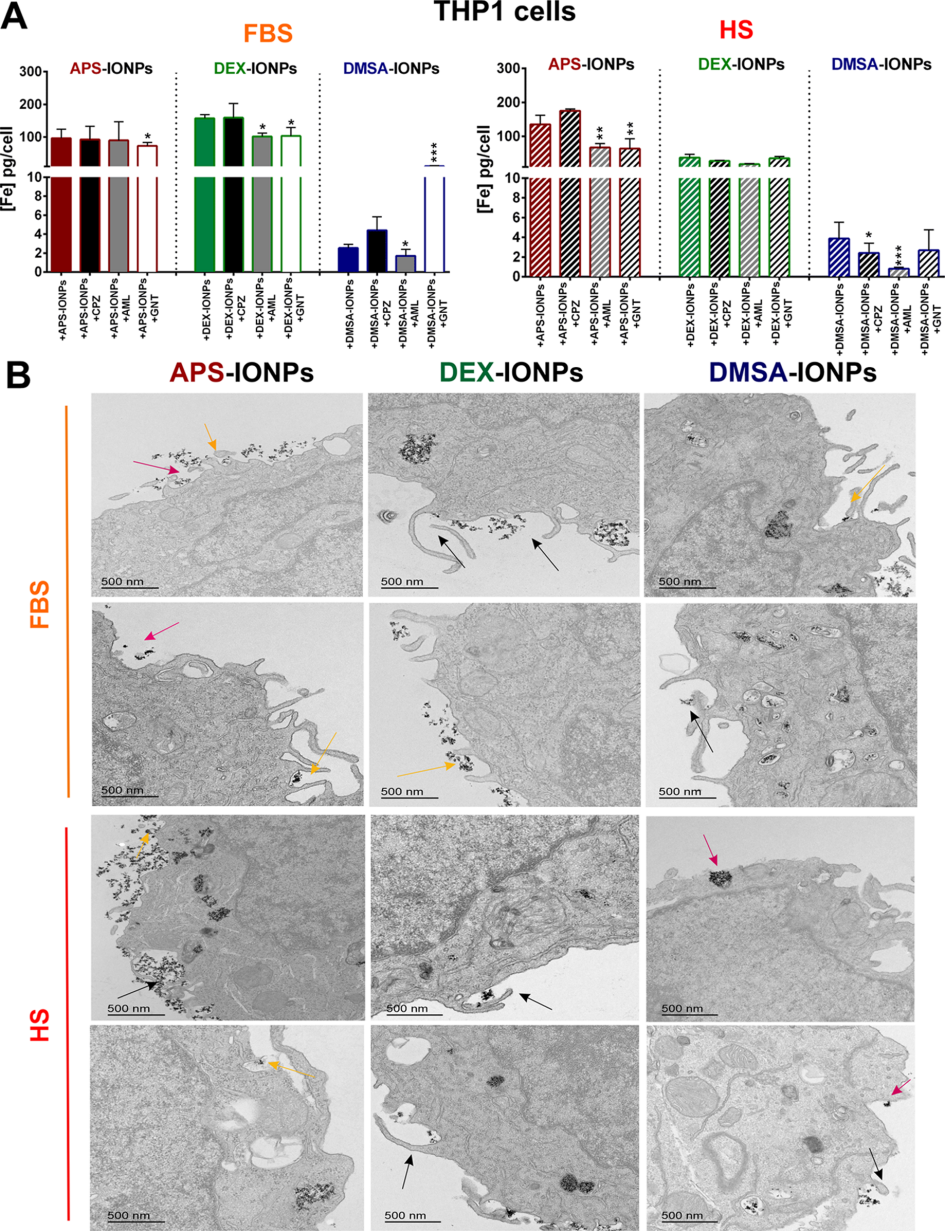
Internalization of IONPs with different biological identities by human THP1 cells. (A) Assay of the endocytic pathways used by APS-, DEX-, and DMSA-IONPs to enter THP1 cells. Specific chemical inhibitors were used to block some of the main pathways of internalization: chlorpromazine (CPZ) to inhibit clathrin-mediated endocytosis; amiloride (AML) to inhibit micropinocytosis; and genistein (GNT) to inhibit caveolae-dependent endocytosis. (B) TEM analysis of the typical membrane structures associated with IONP internalization in medium with FBS (left) or HS (right). Colored arrows indicate the structures associated with IONP internalization: clathrin-mediated endocytosis (clathrin-coated pits, pink); caveolae-mediated endocytosis (flask-shaped structures, yellow); macropinocytosis (macropinosomes, black) in this case THP1 cells were treated with IONPs but in the absence of endocytosis inhibitors. Scale bar: 500 nm. The data are shown as the mean (±SD) of three independent experiments, analyzed with a two-way analysis of variance (ANOVA) and Tukey’s multiple comparisons test: *p < 0.05, **p < 0.01, and ***p < 0.001.

In human THP1 cells, APS-IONPs appeared to preferentially use caveolae-dependent endocytosis when maintained in FBS-supplemented medium, as exposure to genistein reduced their iron content (73.0 ± 10.3 pg/cell) relative to untreated cells (96.4 ± 27.3 pg/cell). However, the iron content of THP1 cells was also reduced by genistein when the IONPs were maintained in HS-supplemented medium (63.7 ± 19.28 pg/cell), and a similar reduction in iron content was also detected when the cells were exposed to amiloride (67.0 ± 11.7 pg/cell) relative to the untreated cells (135.2 ± 26.5 pg/cell), indicating that macropinocytosis is also involved in APS-IONP internalization when the PC was derived from HS (Figure 4A,B). The DEX-IONPs seemed to be internalized preferentially through a macropinocytosis and caveolae-dependent endocytosis when they had been maintained in FBS-supplemented medium, with exposure to amiloride (101.6 ± 10.4 pg/cell) or genistein (103.1 ± 26.0 pg/cell) reducing the iron content in THP1 cells. A similar trend was observed in HS-supplemented medium, suggesting that both the FBS and HS-derived PC facilitate DEX-IONPs internalization through similar pathways. Finally, DMSA-IONPs maintained in FBS-supplemented medium were mainly taken-up by THP1 cells through macropinocytosis (1.7 ± 0.6 pg/cell), while when prepared HS-supplemented medium they were internalized mainly by clathrin-mediated endocytosis (2.4 ± 0.9 pg/cell). In both cases a reduction of iron content was observed when the cells were exposed to the endocytosis inhibitor in FBS- (2.5 ± 0.4 pg/cell) or HS-supplemented medium (3.9 ± 1.6 pg/cell: Figure 4A,B).

TEM images of both the mouse and the human cells were analyzed to corroborate the pathways by which each IONP was internalized in the conditions described above. We identified the endocytic pathways through the ultrastructural morphology of the endocytotic intermediates at the plasma membrane and included the clathrin-coated pits (associated with clathrin-mediated endocytosis), the flask-shaped structures without an electron-dense coat (associated with caveolae-mediated endocytosis), and the larger macropinocytotic vesicles (macropinosomes) that identify macropinocytosis^6^ (Figures 3B and 4B).

Noteworthy, although we detected some inhibition of iron internalization upon treatment with endocytosis inhibitors, an important quantity of IONPs can still enter RAW264.7 and THP1 cells regardless of the PC. Such a fact might be related to the predominance of phagocytosis as the main route for the engulfment of large particles by monocyte/macrophages.^66^ Importantly, phagocytosis and macropinocytosis share similar structural features such as the initial membrane protrusion or ruffles that coalesce into large vacuoles and both processes deeply depend on actin rearrangement, making it difficult to distinguish both mechanisms merely by TEM images.^67^ The main difference relies on the dependence of phagocytosis on phagocytic receptors that recognize opsonized particles.^68^ Since a general phagocytosis inhibitors target actin arrangement, *e.g.*, cytochalasin D, that can also affect macropinocytosis, the use of these inhibitors would not differentiate between these two processes. Amiloride, however, appears to target more specifically micropinocytosis as it lowers the submembranous pH through disturbing the Na^+^/H^+^ exchange.^69^ We, nevertheless, acknowledge the importance that future study addresses how PC nature modulates phagocytosis by macrophages.

The uptake of nanomaterials by macrophages can produce a remarkable increase in TNFα and nitric oxide (NO), considered molecular markers for the immunotoxicity of nanomaterials.^70^ The NP-associated PC appears to promote the release of pro-inflammatory cytokines, as demonstrated elsewhere.^71^ Curiously, a change in the secondary structure of HS albumin bound to NPs could switch the method of cell uptake from albumin receptor-mediated to scavenger receptor-mediated internalization, activating the NF-kB signaling pathway. In addition, IONP PC complexes proved to activate the complement system through classical, lectin-driven, or alternative pathways.^35^ For example, it was demonstrated that DEX-IONP cores incubated in HS or plasma are rapidly opsonized with the third component of complement (C3) through the alternative pathway.^72^ Therefore, we sought to determine the biological impact of IONP internalization on macrophages.

### Influence of the Biological Identity of IONPs on Macrophage Phenotype

Most previous studies that analyzed how the biological identity influences immune cell activation, mainly macrophage activation, focused on the physicochemical features of the IONPs rather than the origin of the PC. Macrophages are critical cells in the immune system that polarize to a classic pro-inflammatory (M1) or anti-inflammatory (M2) phenotype.^73,74^ Thus, we analyzed how the PC associated with IONPs affects the phenotype and functionality of macrophages based on its biological origin. Both the murine RAW264.7^75^ and human THP1^76^ cells adopt a monocyte-like morphology with an undifferentiated phenotype, representing excellent cell models to study macrophage differentiation. Thus, we used the same models as those to assess internalization to analyze macrophage differentiation, exposing the cells to IONPs (125 μg Fe/mL) prepared in MS- or HS-supplemented DMEM (mouse and human model, respectively). We assessed the immunomodulatory influence of the PC-coated IONPs on macrophages by evaluating the expression of the co-stimulatory cell surface markers CD80 and CD86.

**Figure 5:**
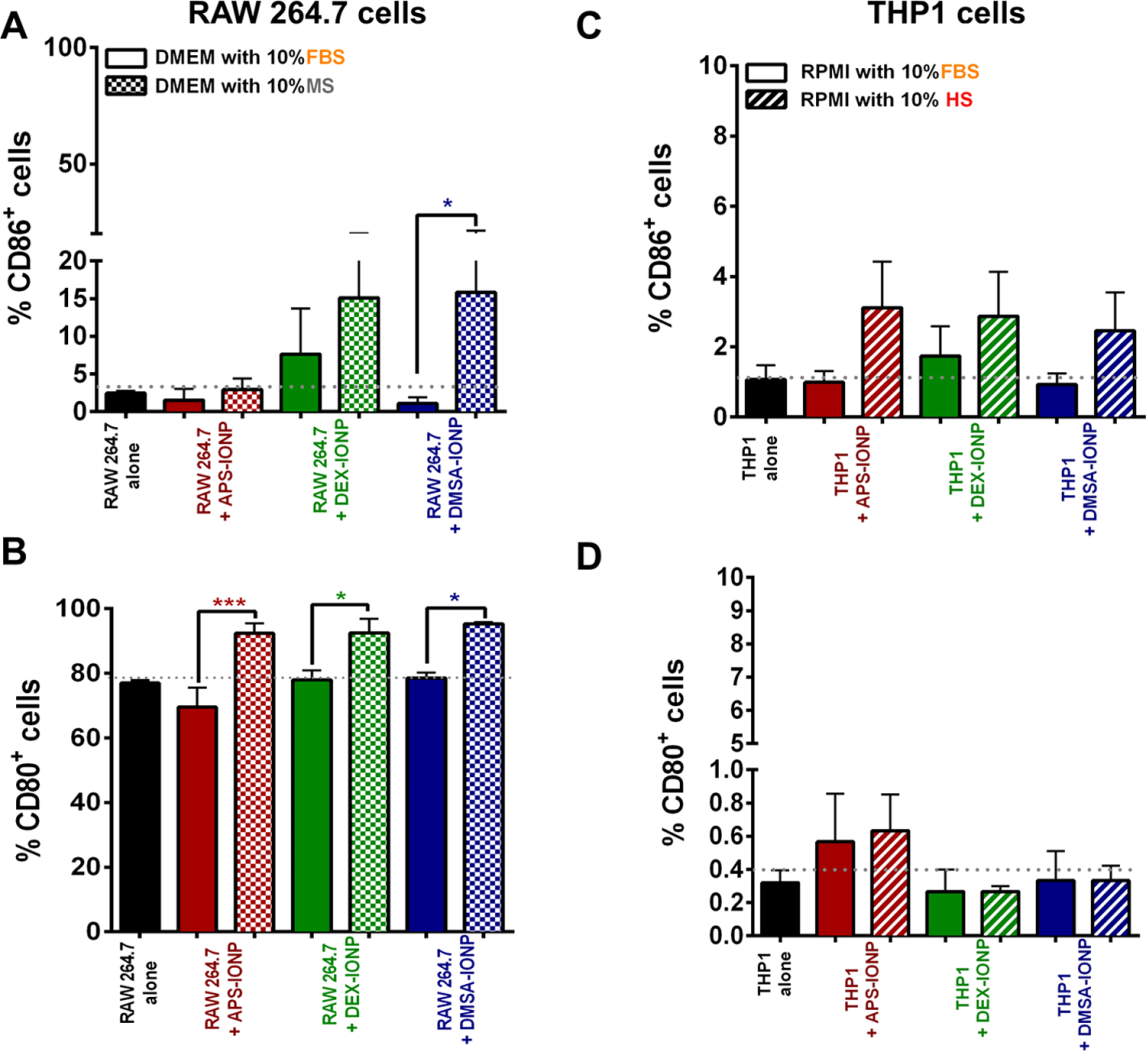
Influence of the PC on macrophage activation. (A, C) Expression of CD86 was determined by flow cytometry. (B, D) CD80 expression was determined by flow cytometry. The RAW264.7 cells were incubated in medium supplemented with 10% FBS or 10% MS, while the THP1 cells were incubated in medium with 10% FBS or 10% HS. The data (mean ± SD) are representative of five independent experiments and analyzed with a Student’s *t*-test: *p < 0.05, **p < 0.01, ***p < 0.001, and ****p < 0.0001.

The DMSA-IONPs induced the expression of CD86, the marker of activation, in RAW267.4 cells maintained in MS-supplemented (15.8 ± 9.6%) relative to the IONPs prepared in FBS-supplemented medium (1.1 ± 0.3%: Figure 5A). Although a similar trend was observed for DEX-IONPs, there was no significant increase in CD86-expressing cells treated with DEX-IONPs prepared in MS (15.1 ± 8.9%) relative to those prepared in FBS-supplemented medium (7.6 ± 1.5%). Moreover, APS-IONPs did not induce CD86 expression in any condition. Notably, all IONPs prepared in MS-supplemented medium promoted the expression of the CD80 marker of activation relative to those IONPs prepared in FBS-supplemented medium (Figure 5B). Together, these data indicate that some proteins in the PC derived from MS activate murine macrophages.

Although there were no significant differences, we observed a decrease in the proportion of CD86^+^ THP1 cells when they were incubated with all of the IONPs in FBS-supplemented medium as opposed to HS-supplemented medium (Figure 5C). Regarding the expression of CD80^+^ THP1 cells, no differences were observed between FBS-supplemented medium and HS-supplemented medium (Figure 5D). Therefore, unlike the murine model in which the MS-derived PC activated RAW264.7 cells, in the human model the FBS and HS-derived PC the differences are very slight, causing similar expression regardless of PC (Figure S8).

**Figure 6:**
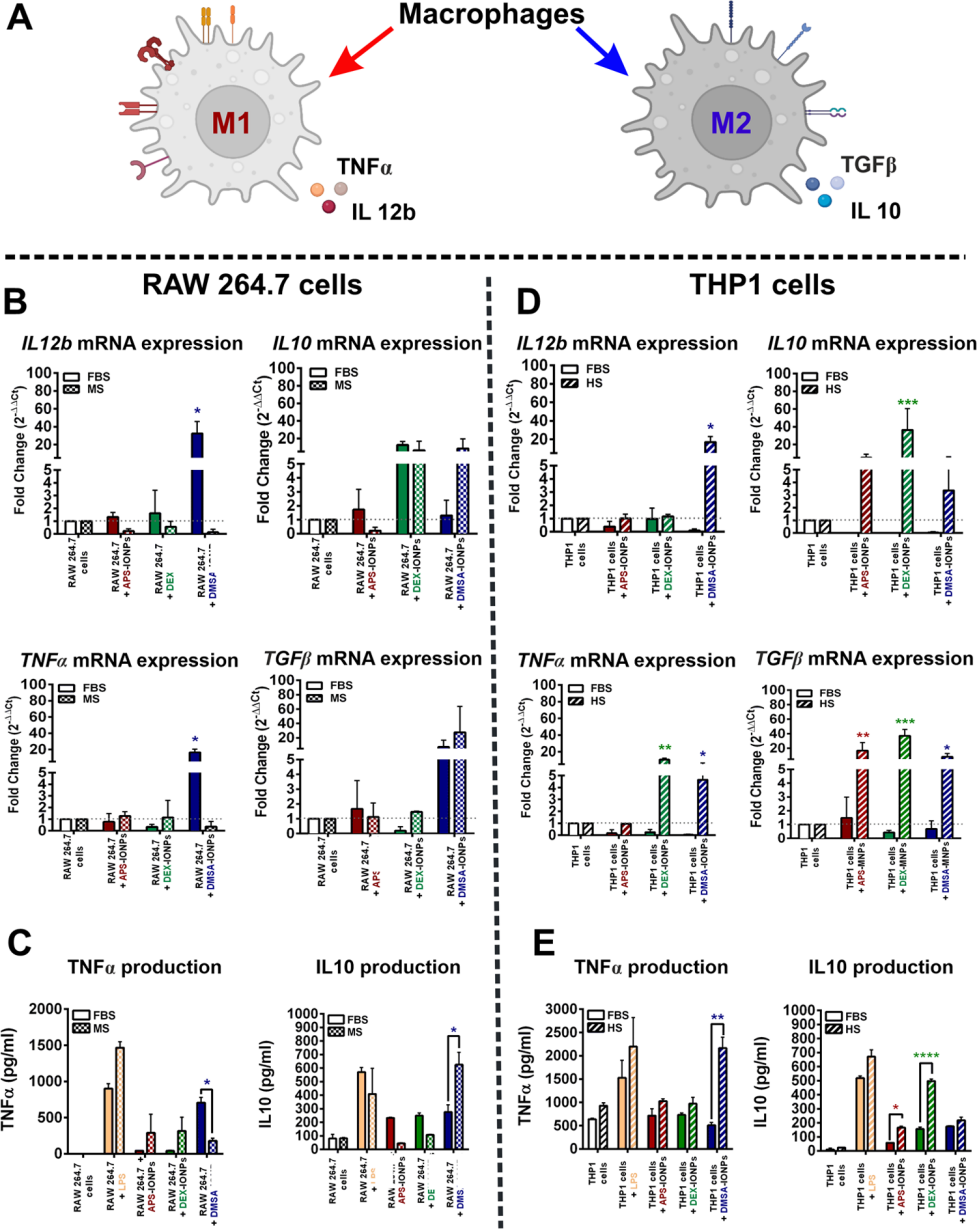
Influence of the protein corona associated with IONPs on the polarization of macrophages. (A) Scheme of macrophage polarization. (B) Expression of the genes involved in M1 (right) and M2 (left) macrophage cell polarization analyzed by real-time quantitative PCR (rt-qPCR) on RNA extracted from RAW264.7 cells exposed to IONPs in medium with different biological serum. (C) TNFα and IL10 production was determined by ELISA in RAW264.7 cells exposed to IONPs in medium with different types of serum. (D) Expression of the genes involved in M1 (right) and M2 (left) macrophage polarization analyzed by rt-qPCR on RNA extracted from THP1 cells exposed to IONPs in medium with different biological serum. (E) TNFα and IL10 production was determined by ELISA in THP1 cells exposed to IONPs prepared in medium with different types of serum. The data (mean ± SD) are representative of three independent experiments and analyzed with a two-way analysis of variance (ANOVA) and Tukey’s multiple comparisons test: *p < 0.05, **p < 0.01, ***p < 0.001, and ****p < 0.0001.

In addition, we determined the macrophage phenotype induced by IONPs (M1/M2) in the same models (Figure 6A), profiling the differentiation markers for M1 (TNFα and IL-12) and M2 (IL-10 and TGF-β) macrophages.^19^ Curiously, semiquantitative polymerase chain reaction (PCR) analysis identified an increase in the M1-associated transcripts (*Il*12*b* 32.7-fold change and *Tnf*α 16.5-fold change) in RAW264.7 cells when exposed to DMSA-IONP in FBS as opposed to MS-supplemented medium (*Il*12*b* 0.13-fold change and *Tnfa* 0.4-fold change). However, we did not detect such an effect when the cells were exposed to IONPs prepared in MS-supplemented medium (Figure 6B, left). Furthermore, the M2-associated mRNA transcripts, *Il*10 and *Tgfb*, tended to shift in the opposite direction, as DMSA-IONPs appeared to promote an M2-like phenotype in MS-supplemented medium (Figure 6B, right). Notably, the DEX-IONPs seemed to increase the *Il*10 transcripts irrespective of the serum source. These results are corroborated by the decrease in TNFα and increase in IL-10 released into the medium when RAW264.7 cells were exposed to DMSA-IONPs in MS relative to FBS-supplemented medium (Figure 6C). No significant changes were detected in cells exposed to APS- or DEX-IONPs. Thus, a DMSA-IONP self identity (MS-derived PC) seemed to promote the differentiation of murine macrophages to an M2-like phenotype, whereas non-self identity (FBS-derived PC) appeared to promote more of an M1-like phenotype.

In the human cell model, we observed the opposite effect on M1 markers. While neither APS- nor DEX-IONPs altered *IL*12*b* expression irrespective of the serum supplement, the DMSA-IONPs significantly enhanced *IL*12*b* mRNA expression in medium supplemented with HS (16.9-fold change) relative to FBS-supplemented medium (0.1-fold change: Figure 6D, left). Similarly, the *TNF*α transcripts increased in THP1 cells exposed to DMSA-IONP in HS-supplemented medium (4.6-fold change) relative to the FBS-supplemented medium (0.04-fold change: Figure 6D, left). This effect on the *TNF*α transcripts was also noted in THP1 cells exposed to DEX-IONPs in HS-supplemented medium (10.6-fold change) relative to FBS-supplemented medium (0.25-fold change: Figure 6D, left). Furthermore, the increase in the TNFα released into the HS-supplemented medium after exposure to DMSA-IONPs, and to a lesser extent APS-IONP- and DEX-IONPs, corroborates the pro-M1 phenotype shift promoted by the HS-derived PC (Figure 6E). Nevertheless, we also detected an increase in the M2-associated transcripts encoding *IL*10 and *TGF*β in HS-derived PC-coated IONPs, independent of the IONP surface nature, indicating that HS-derived PCs promoted a mixed phenotype of activated THP1 cells (Figure 6D, *right*). Consequently, we also detected a trend toward more TNFα and IL10 release into the medium when THP1 cells were exposed to IONPs in HS-supplemented medium (Figure 6E). Together, the human self identity of all IONPs studied seemed to drive a predominantly M2-like phenotype in THP1 cells, whereas THP1 cells remained undifferentiated when confronted with non-self IONPs. The overall M2-like phenotype induced by the self IONPs in both the murine (DMSA-IONPs) and human (all IONPs) models suggests a mechanism that counteracts a profound inflammatory response to the inorganic NPs.

Among the 10 most strongly represented proteins in the MS-derived PCs was Mug1/2, which can regulate the migratory behavior of macrophages and other cells from the innate immune system due to its protease inhibitory activity.^50^ Mugs inhibit thrombin, plasmin, pancreatic elastase, and neutrophil elastase,^77^ and although it was thought Mug was typically a protease inhibitor active in plasma, it is now accepted that it can also act locally and inhibit the migration of several cell types. Taking into account that the serine A3K protease inhibitor, another protease inhibitor, also has anti-inflammatory effects, counteracting some pro-inflammatory scenarios,^51^ the MS-derived PC deposited on the IONPs may produce anti-inflammatory effects, as further supported by the presence of two other well-known anti-inflammatory proteins: ApoA-I^52^ and the heme-scavenger HPX.^53^ As a matter of fact, HPX reverts the pro-inflammatory phenotype of macrophages from M1 to M2 in a model of sickle cell disease, inducing IL-10 secretion and the expression of M2 marker CD206,^53^ resembling what we observed in the self identity mouse model (Figure 6B,C).

In the human self identity model, there was a somewhat mixed M1/M2 phenotype, reflecting the balance among the pro- and anti-inflammatory proteins in the top 10 overrepresented species. Some of these proteins can exert anti-inflammatory activity, such as haptoglobin, which in concert with hemoglobulin, triggers CD163-mediated macrophage activation toward an M2 phenotype,^54^ or promotes the M2 phenotype by increasing CD206 in microglia in an ischemic brain damage model *in vivo.*(78) In addition, AAT can promote an anti-inflammatory environment in some autoimmune diseases^55,56^ antagonizing proteases such as ADAM-17, and attenuating the activation of macrophage/microglia by diminishing MHC class II promoter activity and the expression of pro-inflammatory genes, such as IL1-1b and the endoplasmic reticulum stress marker *ATF*3.^55^ We also perceived ApoA1 enrichment in the HS-derived PCs, which might contribute to its anti-inflammatory activity.^52^

By contrast, α2M can drive macrophage activation toward a pro-inflammatory phenotype through its cognate LRP1/CD91 receptor.^48,57,79^ Nonetheless, α2M can bind to several growth factors and cytokines, probably decreasing their half-life.^80^ Since monocytes and macrophages express α2M receptors, several functional consequences have been described in these cells. For instance, α2M enhances the phagocytic and anti-microbial capacity of macrophages against *Trypanosoma cruzi*,^81^ or it can promote antigen presentation to T cells,^82^ indicating strong potential as an adjuvant. Therefore, global immune regulation by the HS-derived PC derives from a balance of all of these putative effects.

### Influence of IONP Biological Identity on Macrophage Migration

Since macrophage polarization can influence cell migration^19^ and we found many protease inhibitors overrepresented in some PCs, we assessed whether exposure to IONPs affects macrophage migration as a function of their biological identity. Wound closure assays were set up, a standard approach to assess the inhibition of cell migration in two-dimensional (2D) cell cultures, using only murine RAW264.7 cells as the THP1 cell line is an undifferentiated human monocyte cell line that adheres poorly to the surface of the culture dish. A scratch was made in the confluent RAW264.7 cell cultures that were then treated with IONPs (125 μg Fe/mL) for 24 h in FBS-supplemented (non-self) or MS-supplemented (self) DMEM. A normalized directional migration index (DMI) was then obtained by dividing the % DMI of IONP-treated cells by the % DMI of untreated cells grown in the same serum given that RAW264.7 cells cultured in different serum-supplemented media migrate distinctly (Figure S9).

**Figure 7:**
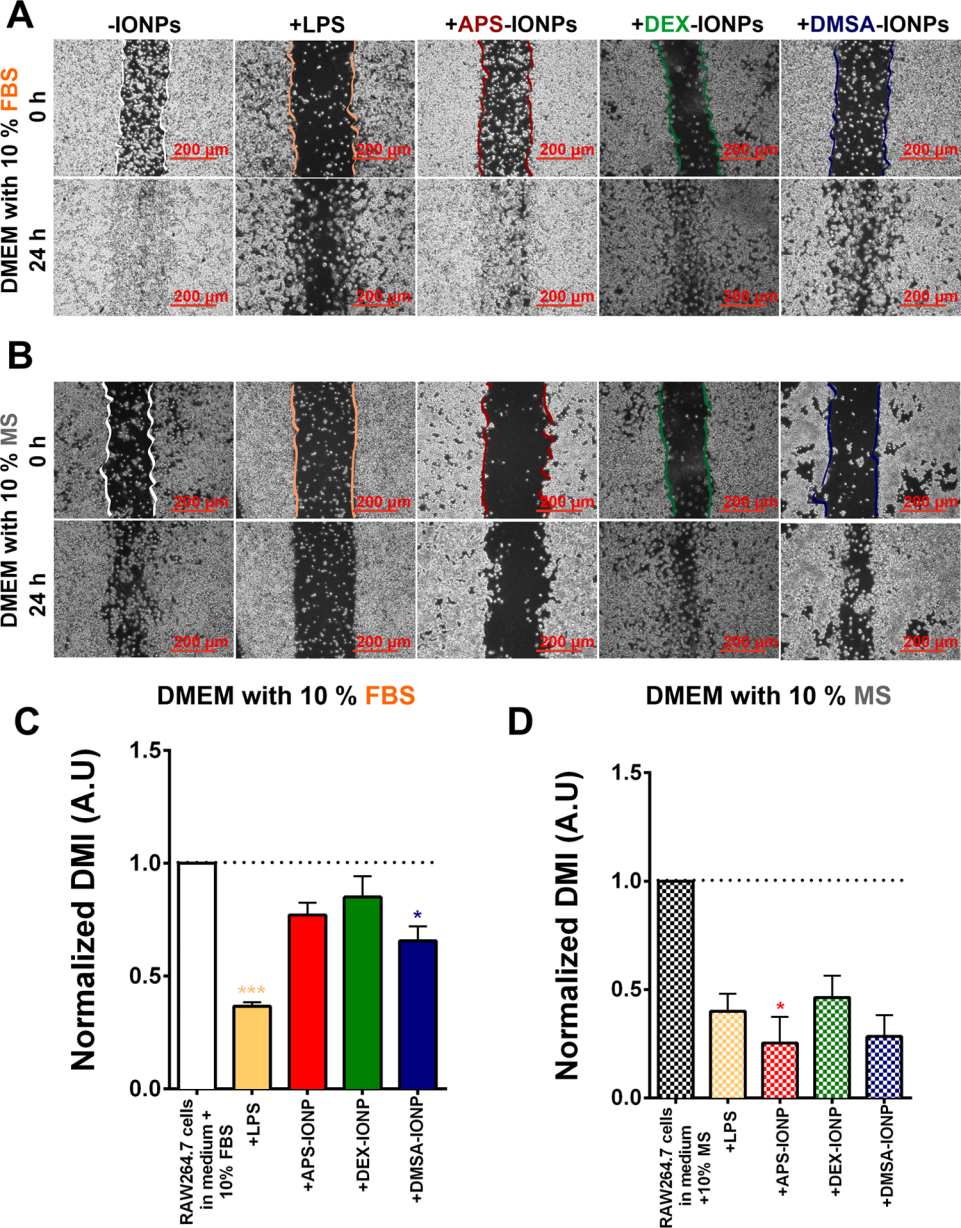
Influence of the protein corona associated with IONPs on macrophage migration. (A, B) Representative images of RAW264.7 cell migration in the *in vitro* scratch wound assays when cultured in different sera after exposure to lipopolysaccharide (LPS) or IONPs. Scale bar: 200 μm. (C, D) Normalized directional migration index was obtained by dividing the % DMI of IONP-treated cells by the % DMI of untreated cells grown in the same serum, and the DMI was measured using ImageJ software from three independent experiments. One-way analysis of variance (ANOVA) and a Student’s t-test were used to assess the data, and the asterisks indicate significant differences: *p < 0.05, **p < 0.01, and ***p < 0.001.

In all cases, self IONPs decreased the relative DMI (APS-IONPS to 0.25 ± 0.12; DEX-IONPs to 0.46 ± 0.12; and DMSA-IONPs to 0.28 ± 0.09), indicating that they inhibited macrophage migration more than the non-self IONPs (APS-IONPS to 0.77 ± 0.05; DEX-IONPs to 0.85 ± 0.09; and DMSA-IONPs to 0.65 ± 0.06: Figure 7). Thus, a self biological identity of IONPs appeared to promote stronger inhibition of murine macrophage migration than a non-self biological identity. The polarization states of macrophages cannot explain their migratory cell behavior on exposure to IONPs, as M2 macrophages are often associated with a high rate of migration or a rate even higher than that of M1 macrophages.^83^ Indeed, the solid 2D adhesion of M1 macrophages correlates with poor or negligible mobility through a three-dimensional (3D) matrix, while the rather moderate or weak adhesion of M2 macrophages favors dynamic cell motility. Such differences arise from the differential expression of the α_D_β_2_ and α_M_β_2_ integrins strongly expressed by M1 macrophages but only moderately by their M2 counterparts.^84^ However, the presence of protease inhibitors as part of the MS-derived PC may further explain why these PCs inhibit macrophage migration in the murine self identity model. Indeed, Mug1/2 and A3K can regulate the migratory behavior of macrophages and other cells in the innate immune system through their protease inhibitor activity.^50,51^

### Influence of IONP Biological Identity on Macrophage ROS Production

Another important molecular signal involved in macrophage polarization is oxidative stress through the production of ROS, known to regulate several molecular pathways and implicated different pathologies.^85^ ROS also represent a major toxicological paradigm of nanomaterials, as many inorganic metallic NPs, like IONPs, induce ROS production.^86^ Therefore, we assessed the levels of ROS induced by the IONPs with different biological identities through a fluorescent Dihydrorhodamine 123 (DHR) assay, interrogating the same systems as before.

**Figure 8:**
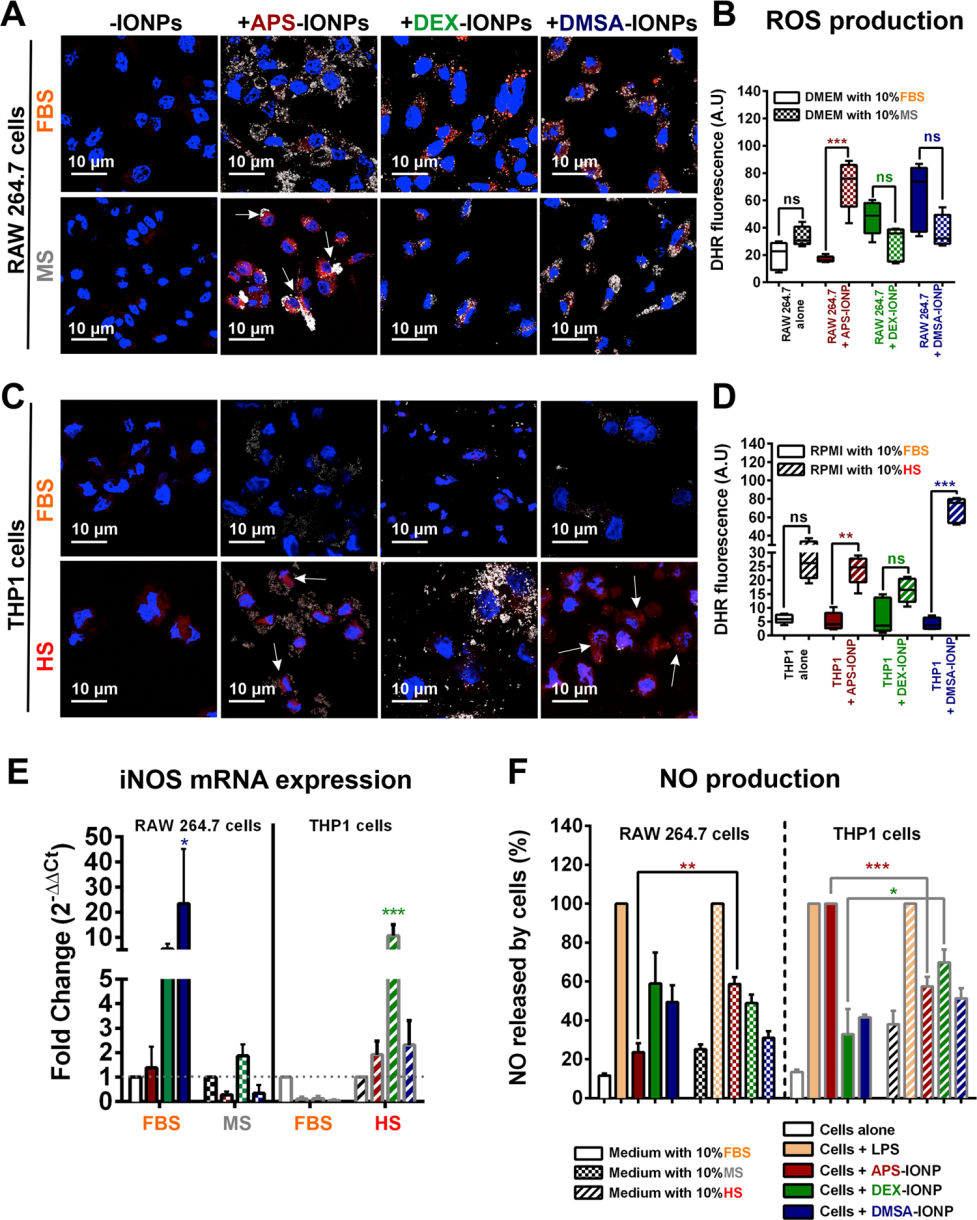
Influence of the protein corona on ROS induction by macrophages. (A, C) ROS generation observed by DHR fluorescence in RAW264.7 or THP1 cells incubated in media supplemented with different sera and exposed to APS-, DEX-, or DMSA-IONPs. Images were taken with a 63× oil objective under a 3× zoom. Scale bar: 10 μm. (B, D) Quantitative image analysis of DHR fluorescence intensity using ImageJ software for both macrophage cell lines. (E) Expression of iNOS measured by RT-qPCR in macrophage cells incubated in media supplemented with different sera and exposed to APS-, DEX-, or DMSA-IONPs. (F) NO production in the two types of macrophage lines incubated in different biological sera. The data (mean ± SD) are representative of three independent experiments and were analyzed with a two-way analysis of variance (ANOVA) and Tukey’s multiple comparisons test: *p < 0.05, **p < 0.01, and ***p < 0.001.

In the murine RAW264.7 cells, the self biological identity of APS-IONPs induced significant ROS accumulation (71.7 ± 7.9) relative to untreated cells (33.6 ± 3.1). Notably, ROS induction was not observed in the non-self model in which exposure to APS-IONPs in FBS-supplemented medium did not increase the amount of ROS (16.9 ± 1.0) relative to untreated cells (19.7 ± 4.5: Figure 8A,B). For the other IONPs, a self biological identity did not enhance ROS production. Nonetheless, we observed a significant increase in ROS production in the non-self biological identity model for DEX- (47.4 ± 5.4) and DMSA-IONPs (63.1 ± 10.8) relative to the untreated cells (19.7 ± 4.5: Figure 8A,B). Accordingly, we also detected an increase in *Nos*2 mRNA transcripts and NO production in RAW264.7 cells exposed to DEX- and DMSA-IONPs in FBS-supplemented medium (Figure 8E,F). Conversely, NO production by RAW264.7 cells exposed to APS-IONPs in MS-supplemented medium was significantly higher than that in cells exposed to DEX- or DMSA-IONPs (Figure 8F). Hence, APS-IONPs promote ROS production when they are of a self biological identity, whereas DEX- and DMSA-IONPs exert a similar effect in a non-self biological situation in the murine model. Such distinctive ROS production may be related to macrophage polarization. M1-polarized macrophages exert strong bactericidal and anti-pathogen activity,^87^ clearing sites of infection by inducing ROS and NO production through NADPH oxidase and NO synthase, respectively.^88^ We found that the non-self biological identity of DMSA-IONPs promoted a robust M1 phenotype in the murine RAW264.7 cell model. By contrast, an apparent M2-like phenotype was evident in the self biological identity model. Therefore, the differences in ROS production might be associated with the polarization of RAW264.7 cells, whereby DMSA-IONPs stimulated ROS production in FBS-supplemented medium but not in MS-supplemented medium. However, APS-IONPs behaved differently, facilitating the production of ROS in the self identity model and as there was no clear phenotype of polarization, this finding cannot be readily explained, suggesting that other mechanisms may well be involved. A similar trend was found for DEX-IONPs that induced ROS production in the non-self biological identity model, even though an M2-like phenotype was confirmed by the increase in the *IL*10 mRNA transcripts.

Recent studies provided evidence that shifts in metabolic reprogramming are involved in macrophage activation. For example, M1-polarized macrophages exhibit enhanced glycolysis, *de novo* fatty acid synthesis, and an exacerbation of the pentose phosphate pathway (PPP) to support pro-inflammatory and microbial killing. By contrast, M2-polarized macrophages augment oxidative phosphorylation and fatty acid oxidation to promote tissue remodeling and repair.^89^ Furthermore, M1-polarized macrophages have a smaller lattice size and a predominantly spherical mitochondrial morphology due to excess mitochondrial fission.^90,91^ Thus, we analyzed macrophage mitochondrial morphology to better understand the impact of the PC on IONP-induced oxidative stress. TEM images of M1-polarized macrophages derived from RAW264.cells exposed to DMSA-IONPs in FBS-supplemented medium revealed mitochondria to have a spherical morphology, suggesting they had suffered fission events (Figure S10A). Conversely, M2-like macrophages generated from RAW264.7 cells that were exposed to self DMSA-IONPs or non-self DEX-IONPs had more elongated mitochondria (Figure S10B). Therefore, mitochondrial morphology provided further evidence of the M1 and M2 macrophage features generated in accordance with the self and non-self biological identity of the IONPs.

In the human THP1 cell model, we observed no ROS induction by any non-self IONP (FBS-supplemented RPMI), consistent with the virtually unchanged THP1 polarization and *Nos*2 mRNA expression in this state (Figure 8C–E). However, self DMSA-IONPs (HS-supplemented RPMI) promoted a significant increase in ROS, consistent with the robust mixed M1/M2 polarization and the elevated *Nos*2 mRNA expression (Figure 8C–E). Notably, the THP1 cells exposed to non-self APS-IONPs in FBS-supplemented medium secreted a large quantity of NO even though there was no evident increase in *Nos*2 mRNA expression, suggesting the involvement of other molecular mechanisms (Figure 8E,F).

Noteworthy, IONP-induced ROS production not only relies on macrophage phenotype but also other nanoparticle-related parameters. Since IONP-triggered ROS production depends on the balance of the different atomic iron species that contribute to the labile iron pool of the cell and the redox state of the cell, the IONP degradation kinetic would also affect the extent of ROS level. Consequently, IONPs can modulate macrophage biology through the exacerbation of oxidative stress.^92^ Indeed, we have previously demonstrated that DMSA-IONP and APS-IONP transit differently inside RAW264.7 macrophages whereby APS-IONPs are quickly concentrated in phagolysosomes while DMSA-IONPs delay their accumulation. Such a distinctive intracellular transit might likely promote different coating and iron oxide core degradation leading to a different atomic iron level in a spaciotemporal basis. We have also demonstrated differences in IONP metabolization *in vivo*, whereby macrophages of diverse phenotype metabolize IONP in different extents through the induction of iron metabolism and iron recycling factors.^93^

In summary, we observed a clear correlation between ROS production and macrophage polarization of cells exposed to DMSA-IONPs, although this was less evident for APS- and DEX-IONPs. Therefore, the biological identity of IONPs derived from the biological serum may be a critical parameter when studying the response of macrophages to IONPs, both *in vitro* and *in vivo*.

## Conclusions

We investigated the differences in PC composition (biological identity) deposited on IONPs in different sera among those commonly used *in vitro* and *in vivo*. We defined a self biological identity (cells and serum from the same species) and a non-self biological identity (cells and serum from different species) to understand how the PC of the IONPs interacted with macrophages. Comprehensive proteomic approaches demonstrated that the profile of the PC differs according to the biological serum but not so much due to the coating, indicating that the biological identity of IONPs is highly dependent on the physiological environment. While in the FBS-derived PC α-2-HS-glycoprotein, antithrombin-III and albumin prevailed, MS-derived PCs contain serotransferrin, and the anti-inflammatory ApoA-I and Mug1 proteins. By contrast, the HS-derived PCs appeared to acquire both anti-inflammatory (haptoglobin, AA-I, ApoB-100, and AAT) and pro-inflammatory proteins (α2M). The distinct biological identities of the IONPs also influenced macrophage activation and polarization. However, the effect on the functional aspects of macrophages was more evident for DMSA-IONPs, with self DMSA-IONPs promoting activation and M2 polarization of murine macrophages, while their non-self biological identity favor M1 polarization. Consequently, M1-polarized macrophages produce larger quantities of ROS. In the human context, we observed the opposite effect, whereby self DMSA-IONPs promote a mixed M1/M2 polarization with an increase in ROS production and non-self DMSA-IONPs produce stealthy NPs with no evident effect on human macrophages. However, further analysis should be done in the future in a more complex environment, *i.e., in vivo*, to consider other factors such as IONP biodistribution, microenvironment pH and redox conditions, and cellular components, and how they can affect IONP-macrophage interaction. Together, we provide evidence that the biological identity of IONPs strongly affects their interaction with macrophages and ultimately, it defines their biological impact on the immune system.

## Materials and Methods

### IONP Synthesis and Characterization

IONPs were obtained by co-precipitation^26^ of a mixture of Fe(II) and Fe(III) salts in aqueous media, the iron oxide cores were then coated with APS, DEX, or DMSA.^6^ The IONP’s particle size and shape were characterized by transmission electron microscopy (TEM, 100 keV JEOL microscope), and their colloidal properties were assessed by DLS with a Zetasizer Nano S apparatus (Malvern Instruments) and inductively coupled plasma-optical emission spectrometry (ICP-OES). TEM samples were prepared by placing one drop of a dilute IONP suspension in water on a carbon-coated copper grid and allowing the solvent to evaporate slowly at room temperature (RT). The mean particle size and distribution were evaluated by measuring at least 250 particles.

### Analysis of the Colloidal Status of IONPs upon PC Formation

The APS-, DEX-, or DMSA-IONPs (125 μg Fe/mL) were assessed after a 24 h incubation at 37 °C in medium supplemented with different biological sera: 10% FBS, MS, or HS. The hydrodynamic size and *Z*-potential of the IONPs incubated with the different serum was measured by DLS.

### Proteomic Profiling of the Protein Corona

The PC was formed on the APS-, DEX-, or DMSA-IONPs by incubating for 24 h at 37 °C in medium supplemented with 10% FBS, MS, or HS at a final volume of 8 mL. The IONPs were then magnetically separated from the medium using a neodymium magnet (with a gradient of 0.1 T/cm), gently washed with PBS, and recovered by centrifugation three times. To extract the proteins from the PC, the IONPs were resuspended in a protein extraction buffer containing: 1%Triton X-100; 1 mM EDTA; and the protease and phosphatase inhibitors 1 μg/mL leupeptin, 5 nM NaF, 1 mM sodium orthovanadate, 1 mM phenylmethylsulphonyl fluoride (PMSF), 1 μg/mL aprotinin, and 1 μg/mL okadaic acid. The total protein concentration of each extract was quantified using the BCA kit (Thermo Fisher) and the PC protein extracts were washed sequentially for 30 min with different buffers (50 mM HEPES, 0.1% *N*-octyl-β-d-glucopyranosid (OGP) [pH 7] or 100 mM sodium acetate, NaAc, 0.1% OGP [pH 5]). Each sample was then shaken in a tube rotator with 2 mL with both buffers mentioned above, for 30 min and then centrifuged for 10 min at 4 °C in a microcentrifuge. The protein samples (10 μg) were loaded individually onto a 12% SDS-PAGE gel and after a short (10–15 min) separation, each sample was cut into 3–4 slices and digested with trypsin using an automatic robot Proteineer (Bruker, Bremen, Germany), following the protocol proposed by Schevchenko et al.^94^ After protein digestion, the peptides were extracted, pooled, dried by speed-vacuum centrifugation and stored at −20 °C prior to nano-liquid chromatography electrospray ionization-tandem mass spectrometric (nano-LC-ESI-MSMS) analysis, which was performed on an Eksigent one-dimensional (1D) nano-high-performance liquid chromatography (nano-HPLC) coupled to a 5600 TripleTOF QTOF mass spectrometer (Sciex, Framingham, MA). The analytical column used was a Waters UPLC silica-based reversed-phase C18 75 μm × 15 cm column (particle size 1.7 μm) and the trap column was an Acclaim PepMap 100 (particle size 5 μm, 100 Å pore size) connected in-line to the analytical column. The loading pump supplied a solution of 0.1% formic acid (FA) in 98% water/2% acetonitrile (ACN: Scharlab, Barcelona, Spain) at 3 μL/min. The gradient pump provided a flow rate of 250 nL/min and was run under gradient elution conditions, using 0.1% FA (Fluka, Buchs, Switzerland) in water as mobile phase A and 0.1% FA in 100% ACN as mobile phase B. Gradient elution was performed according to the following scheme: isocratic conditions of 96% A/4% B for 5 min, a linear rise to 40% B in 105 min, a linear increase to 95% B in 2 min, 95% isocratic conditions of B for 5 min and return to the initial states in 10 min. The injection volume was 5 μL. The LC system was coupled via a nanospray source to the mass spectrometer. Data acquisition was carried out using the dynamic exclusion option to obtain full scan MS spectra (*m*/*z* range 350–1250) followed by collision-induced dissociation (CID) MS tandem spectra corresponding to the 25 most abundant precursor ions. The acquisition time was 250 and 100 ms for the MS and CID tandem MS spectra, respectively. All data were obtained using the Analyst TF 1.7 software (AB SCIEX), and the raw data were converted to mgf format using Peak View v1.2.0.3, and Peaks v7.5 (Bioinformatics Solutions, Waterloo, ON, Canada) was used to search composite target/decoy databases built from *Bos taurus*, *Mus musculus*, or *Homo sapiens* protein entries downloaded from Uniprot Knowledgebase, together with commonly occurring contaminants. The search engine was configured to match potential peptide candidates with a mass error tolerance of 25 ppm and fragment ion tolerance of 0.05 Da, allowing for up to three missed tryptic cleavage sites, considering fixed carbamidomethylation of cysteine and variable oxidation of methionine. The results were filtered for a false discovery rate (FDR) ≤ 0.01 (peptide level) and only proteins with at least one unique peptide were considered.

### Cell Culture

The murine RAW264.7 (ATCC: TIB-71) macrophage cell line was cultured in Dulbecco modified Eagle’s medium (DMEM) supplemented with 10% (v/v) FBS, 100 U/mL penicillin, 100 U/mL streptomycin, 2 mM l-glutamine, and 1 mM sodium pyruvate (all from Biowest). The human THP1 (ATCC TIB-202) monocyte cell line was cultured in Roswell Park Memorial Institute (RPMI) 1640 medium supplemented with 10% (v/v) FBS, 100 U/mL penicillin, 100 U/mL streptomycin, 2 mM l-glutamine, and 1 mM sodium pyruvate (all from Biowest). Both these cell lines were maintained under standard culture conditions: 37 °C, 5% CO_2_, and 90% relative humidity.

### Quantification of Iron Internalization by ICP-OES

Cells were seeded in 6-well plates at a density of 3 × 10^5^ cells per well and cultured for 24 h at 37 °C in medium supplemented with 10% FBS, MS, or HS. APS-, DEX-, or DMSA-IONPs (125 μg Fe/mL) were then added for 24 h, after which the cells were collected, washed three times with PBS, and the number of cells was counted in a Neubauer chamber. The samples were digested in HNO_3_ (1 mL) for 1 h at 90 °C, and the amount of iron per cell was measured by ICP-OES (PerkinElmer-2400).

### Elucidation of the Endocytosis Pathways

Macrophages were seeded in 12-well plates at a density of 2 × 10^4^ cells per well and then preincubated with selective endocytic inhibitors for 2 h at non-toxic concentrations: Amiloride (1 μg/mL), an inhibitor of macropinocytosis; Chlorpromazine (5 μg/mL), an inhibitor of clathrin-mediated endocytosis; and Genistein (25 μg/mL), an inhibitor of caveolae-dependent endocytosis. The cells were then incubated with APS-, DEX-, or DMSA-IONPs (125 μg Fe/mL) for 24 h in the presence of the same dose of the inhibitors and they were then cultured in medium supplemented with 10% FBS, MS, or HS for 24 h at 37 °C. Finally, the total amount of internalized iron was quantified by ICP-OES, as described previously.

### TEM imaging

For TEM, 1 × 10^6^ macrophages were seeded in Petri dishes for 24 h and then exposed for 24 h to an optimal concentration of IONPs resuspended in a medium supplemented with the different biological sera. Non-internalized IONPs were removed by washing with PBS before the cells were fixed at RT in 2% glutaraldehyde and 1% tannic acid diluted in 0.4 M HEPES [pH 7.2]. The cells were then washed and resuspended in HEPES buffer, post-fixed at 4 °C with 1% osmium tetroxide (1 h) and 2% uranyl acetate (30 min), dehydrated in a series of acetone solutions, and gradually infiltrated with Epon resin. The resin was allowed to polymerize at 60 °C for 48 h and ultrathin sections (60–70 nm) were obtained with a diamond knife mounted on a Leica EM UC6 ultramicrotome. The sections were attached to a formvar/carbon-coated gold grid and visualized on a JEOL-1011 transmission electron microscope, acquiring images at different magnifications with a Gatan ES1000Ww camera.

### Flow Cytometry

To analyze surface marker expression, macrophages were treated for 24 h with IONPs in a medium supplemented with different biological sera and they were then stained with: BIOT-anti-mouse CD86 (BIOLEGEND, b128959); PE-anti-mouse CD80 (PARMIGEN, 5533759); PE-anti-human CD86 (IMMUNOTECH, IM2729); and FITC-anti-human CD80 (BIOLEGEND, 305206). The data were acquired on an FC500 flow cytometer and analyzed with the FlowJo software.

### RNA Extraction, Reverse Transcription, and RT-qPCR

The cells were treated for 24 h with APS-, DEX-, or DMSA-IONPs (125 μg Fe/mL) in medium supplemented with one of the different biological sera, and they were then collected for RNA extraction. The total RNA from untreated and treated cells was extracted using the High Pure RNA Isolation Kit (Roche), and quantified with NanoDrop, reverse-transcribing 2 μg of RNA to cDNA using the High-Capacity cDNA Reverse Transcriptase kit (Applied Biosystems, Thermo Fisher) and random primers. This cDNA was used to perform RT-qPCR with the Power SYBR Green PCR mix (Applied Biosystems, Thermo Fisher) and the primers listed in Table S1 (all from Sigma). The RT-qPCR expression data were quantified according to the 2^–ΔΔCt^ formula and normalized to β-actin mRNA.

### Quantification of Pro-Inflammatory and Anti-inflammatory Cytokines

Macrophages were treated for 24 h with APS-, DEX-, or DMSA-IONPs (125 μg Fe/mL) in medium supplemented with the different biological sera, and the supernatant was collected. Cytokine concentrations were measured using commercial ELISA kits according to the manufacturer’s recommendations: Mouse TNF-α DuoSet ELISA DY410-05, Mouse IL-10 DuoSet ELISA DY417-05, Human TNF-α DuoSet ELISA DY210-05, and Human IL-10 DuoSet ELISA DY217B-05. Triplicate samples were analyzed in all cases.

### Migration Assays

A wound-like scratch was induced in confluent cultures of RAW264.7 cells that were then exposed for 24 h to APS-, DEX-, or DMSA-IONPs (125 μg Fe/mL), or lipopolysaccharide (LPS, 5 μg/mL: Sigma), in DMEM supplemented with 10% FBS or MS. Cell migration was then monitored every 30 min over 24 h for wound closure, acquiring images on a Leica inverted fluorescence microscope DMI6000B. The directional rate of migration was calculated as
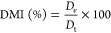
where *D*_v_ is the vectorial distance and *D*_t_ is the total distance.^95^

### Analysis of the Oxidative Stress Induced by IONPs

#### Dihydrorhodamine 123 Staining

The production of ROS was detected by DHR probe staining (Molecular probes, Carlsbad, CA) and analyzed by dark-field confocal microscopy. This nonfluorescent ROS indicator can be oxidized inside cells to the fluorescent rhodamine 123. Accordingly, cells were cultured for 24 h on coverslips in a 24-well plate and in medium supplemented with 10% FBS, MS, or HS, and the medium was removed once the cells were attached to the coverslip before exposing them for 24 h to APS-, DEX-, or DMSA-IONPs (125 μg Fe/mL). The coverslips were then rinsed three times with PBS, and the cells were incubated for a further 30 min with DHR (diluted 1:500 in medium) under cell culture conditions. After 3 washes in PBS, the cells were fixed for 15 min with 4% paraformaldehyde (PFA), stained for 10 min with DAPI (diluted 1:500 in PBS), washed again, and mounted with Fluoromont-G. Images were obtained on a dark-field Leica TCS SP5 confocal microscope with the 63× oil objective and analyzed, quantifying the DHR signal fluorescence intensity with ImageJ software.

#### Nitric Oxide Assay

Macrophages were seeded in 96-well plates (2 × 10^5^ cells/mL, 150 μL/well) and after 24 h, the cells were exposed to LPS (5 μg/mL) and to the different IONPs (125 μg Fe/mL). The NO released by macrophages was then determined using the Griess reagent kit (Thermo Fisher, G-7921) according to the manufacturer’s instructions, expressing the results as the mean (±standard deviation) percentage of NO released relative to the control LPS-activated cells.

### Statistical Analysis

All of the data are presented as the mean (±standard deviation, SD). One-way and Two-way analysis of variance (ANOVA), or Student’s *t*, Sidak’s, and Tukey tests were applied to calculate the significance of the differences between the distinct values. Values of *p* < 0.05 were considered statistically significant, presented as: *p < 0.05, **p < 0.01, ***p < 0.001, and ****p < 0.0001. GraphPad Prism software (version 6.01) was used for the statistical analysis.

## Supporting Information Available

Supplementary Materials and Methods: Study of Corona Formation in different types of biological sera, IONP treatment and toxicity assay, analysis of IONP uptake by macrophage cells, list of primers for real-time quantitative PCR (RT-qPCR); Supplementary Results: Physicochemical characterization of IONPs, analysis of the dynamics of PC formation on IONPs with different coatings according to the species origin of the serum, evaluation of IONPs stability with different coatings according to species of the serum, characterization of the PC on the IONP surface based on the species origin of the serum, IONP toxicity, IONP uptake by macrophage cells in different biological sera, influence of the biological identity of IONPs on macrophage phenotype, influence of the type of biological serum on macrophage migration, and analysis of mitochondrial morphology according to the type of biological serum used after exposure to DMSA-IONPs (PDF)
