# Supplementary material for: Interaction of Iron Oxide Nanoparticles with Macrophages Is Influenced Distinctly by “Self” and “Non-Self” Biological Identities
Source: ACS Appl Mater Interfaces. 2023 Jul 21;15(30):35906–26. doi: 10.1021/acsami.3c05555 (PMC10401511; doi:10.1021/acsami.3c05555)
Supplement: Supplementary file 1 — am3c05555_si_001.pdf [file am3c05555_si_001.pdf]

Supporting Information

The Interaction of Iron oxide Nanoparticles with Macrophages is Influenced Distinctly by 'Self' and 'Non-self' Biological Identities

Yadileiny Portilla [1], Vladimir Mulens-Arias [1#], Neus Daviu [1], Alberto Paradela [2], Sonia Pérez-Yagüe [1] and Domingo F. Barber [1]*

[1]Department of Immunology and Oncology and Nanobiomedicine Initiative, Centro Nacional de Biotecnología (CNB-CSIC), Darwin 3, 28049 Madrid, Spain.

[2]Proteomics Facility, Centro Nacional de Biotecnología (CNB-CSIC), Darwin 3, 28049 Madrid, Spain.

[#]Current address: Stem Cell Biology, Developmental Leukemia and Immunotherapy Laboratory, Josep Carreras Leukemia Research Institute, School of Medicine, Barcelona University, Carrer Casanova 143, 08036 Barcelona, Spain.

Corresponding Author: **E-mail: dfbarber@cnb.csic.es (DFB.)*

SUPPLEMENTARY MATERIALS AND METHODS

Study of Corona Formation in different types of biological sera. To study the process of PC formation, we incubated APS-, DEX- or DMSA-IONPs (125 μg Fe/ml) in DMEM supplemented with a 10% FBS, MS or HS at 37 °C for different times (0, 1, 3, 5, 10, 24, 48 or 72 h). The hydrodynamic size of the IONPs incubated with the different sera was measured by DLS using a NanoSizer ZS (Malvern)[1].

IONP treatment and toxicity assay. Cells were exposed to the IONPs (125 μgFe/ml unless otherwise stated) and their viability was determined with the colorimetric PrestoBlue assay (Invitrogen). The PrestoBlue reagent contains a cell permeable blue and non-fluorescent solution of resazurin that can be metabolized inside the viable cells to a red fluorescent compound called resorufin. This change of color and fluorescence serves as an indicator of cell viability. Cell viability was calculated as the % cell viability = (IONP treated cell fluorescence - mean fluorescence)/ (untreated cell fluorescence - mean fluorescence) × 100.

Analysis of IONP uptake by macrophage cells

Perls' Prussian blue staining. For iron staining, the cells were washed with PBS after incubation with the APS-, DEX- or DMSA-IONPs (125 μgFe/ml) in DMEM or RPMI with 10% of FBS, MS or HS. After IONP treatment, the cells were fixed in 4% paraformaldehyde (PFA) for 15 min, permeabilized with 0.05% Triton X-100 for 5 min, stained with an equal volume of HCl 4% and potassium ferrocyanide trihydrate 4% for 30 min, and counterstained with neutral red 0.5% for 2 min. The cells were then washed with distilled water, air-dried and mounted in medium (7.7% gelatin and 54% glycerol). Images were acquired on an Olympus IX70 inverted bright field microscope with a 63x oil objective.

Confocal microscopy imaging of internalized IONPs. The macrophage cells were grown on poly-lysine coated coverslips for 24 hours and then exposed to APS-, DEX- or DMSA-IONPs (125 μgFe/ml) for 24 h in the different sera. Subsequently, the cells were treated for 2 h at 37 °C with LysoTracker green (1:400 in culture medium: Invitrogen) and then for 30 min at RT with WGA (1:200 in PBS: Invitrogen). The cells were then washed, fixed with 4% PFA (15 min) and then counterstained for 10 min at RT with DAPI (1:500 in PBS: Sigma). Finally, the samples were mounted in Fluoromount G (ThermoFisher) and images were acquired on a confocal multispectral Leica TCS SP5 system, with a 63X/1.4 NA oil objective and a 3X zoom.

List of primers for real-time quantitative PCR (RT-qPCR)

The primers used (all from Sigma) are indicated in **Table S1**.

Gene	Forward (5'-3')	Reverse (5'-3')
Human primers		
IL10	GCCTTTAATAAGCTCCAAGAG	ATCTTCATTGTCATGTAGGC
IL12B	AGAAAGATAGAGTCTTCACGG	AAGATGAGCTATAGTAGCGG
TNFα	AGGCAGTCAGATCATCTTC	TTATCTCTCAGCTCCACG
TGFβ	AACCCACAACGAAATCTATG	CTTTTAACTTGAGCCTCAGC
ACTB	GACGACATGGAGAAAATCTG	ATGATCTGGGTCATCTTCTC
Mouse primers		
il10	CAGGACTTTAAGGGTTACTTG	ATTTTCACAGGGGAGAAATC
il12b	CATCAGGGACATCATCAAAC	CTCTGTCTCCTTCATCTTTTC
tnfα	CTATGTCTCAGCCTCTTCTC	CATTTGGGAACTTCTCATCC
tgfβ	GGATACCAACTATTGCTTCAG	TGTCCAGGCTCCAAATATAG
actb	GATGTATGAAGGCTTTGGTC	TGTGCACTTTTATTGGTCTC

SUPPLEMENTARY RESULTS

Physicochemical characterization of IONPs. The main physicochemical characteristics of the IONPs used in this study are summarized in **Figure S1**, characterizing their hydrodynamic size in water.

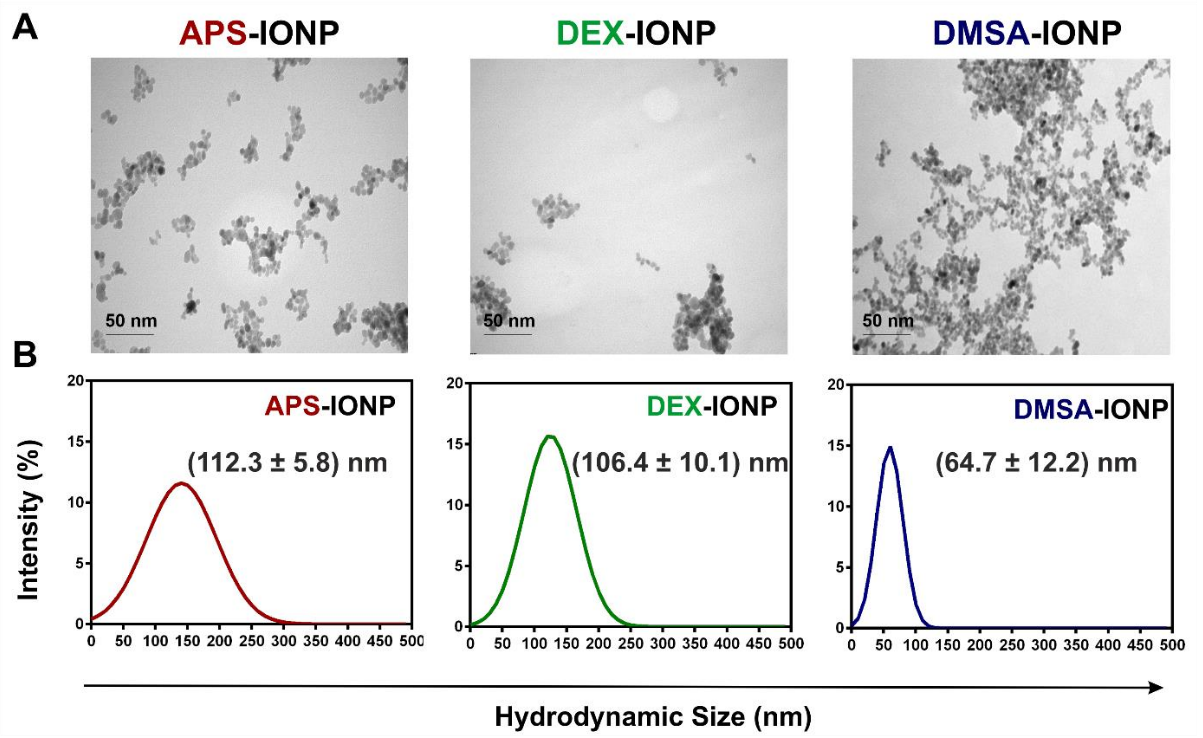

Figure S1. IONP characterization. (A) TEM images of APS-, DEX- and DMSA-coated IONPs, scale bar: 50 nm. (B) Hydrodynamic size of the APS-, DEX- and DMSA-IONPs determined by DLS.

Analysis of the dynamics of PC formation on IONPs with different coatings according to the species origin of the serum. The kinetics of PC formation are shown below, highlighting its stabilization at 24 h regardless of the type of IONP or the biological serum used (**Figure S2**).

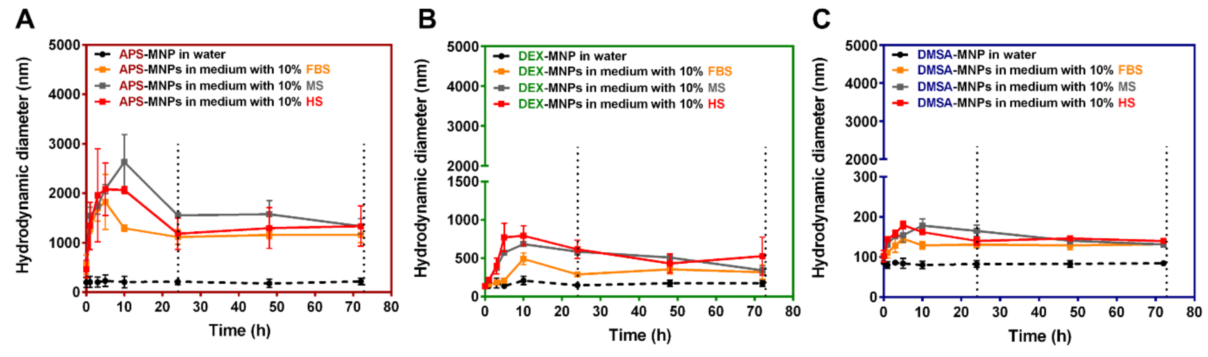

Figure S2. The dynamics of PC formation on APS-, DEX- and DMSA-IONPs in medium supplemented with different biological sera. (A) Hydrodynamic size of APS-IONPs, (B) DEX-IONPs and (C) DMSA-IONPs over time as determined by DLS. DLS measurements were performed in triplicate in all cases.

Evaluation of IONPs stability with different coatings according to species of the serum. The stability of all three IONPs was evaluated by obtaining DLS measurements after the incubation of the APS-, DEX- or DMSA-IONPs in medium alone or in medium (DMEM or RPMI) supplemented with different biological sera for 24 h. In **Figure S3**, there are no signs of agglomeration during the 24 h period as the distribution is homogeneous. We did not detect IONPs sedimentation caused by large agglomerates of IONPs; we did not detect multiple peaks in the hydrodynamic size distributions that would indicate hetero-aggregate formation, as shown below. The results obtained are shown in **Figure S3**.

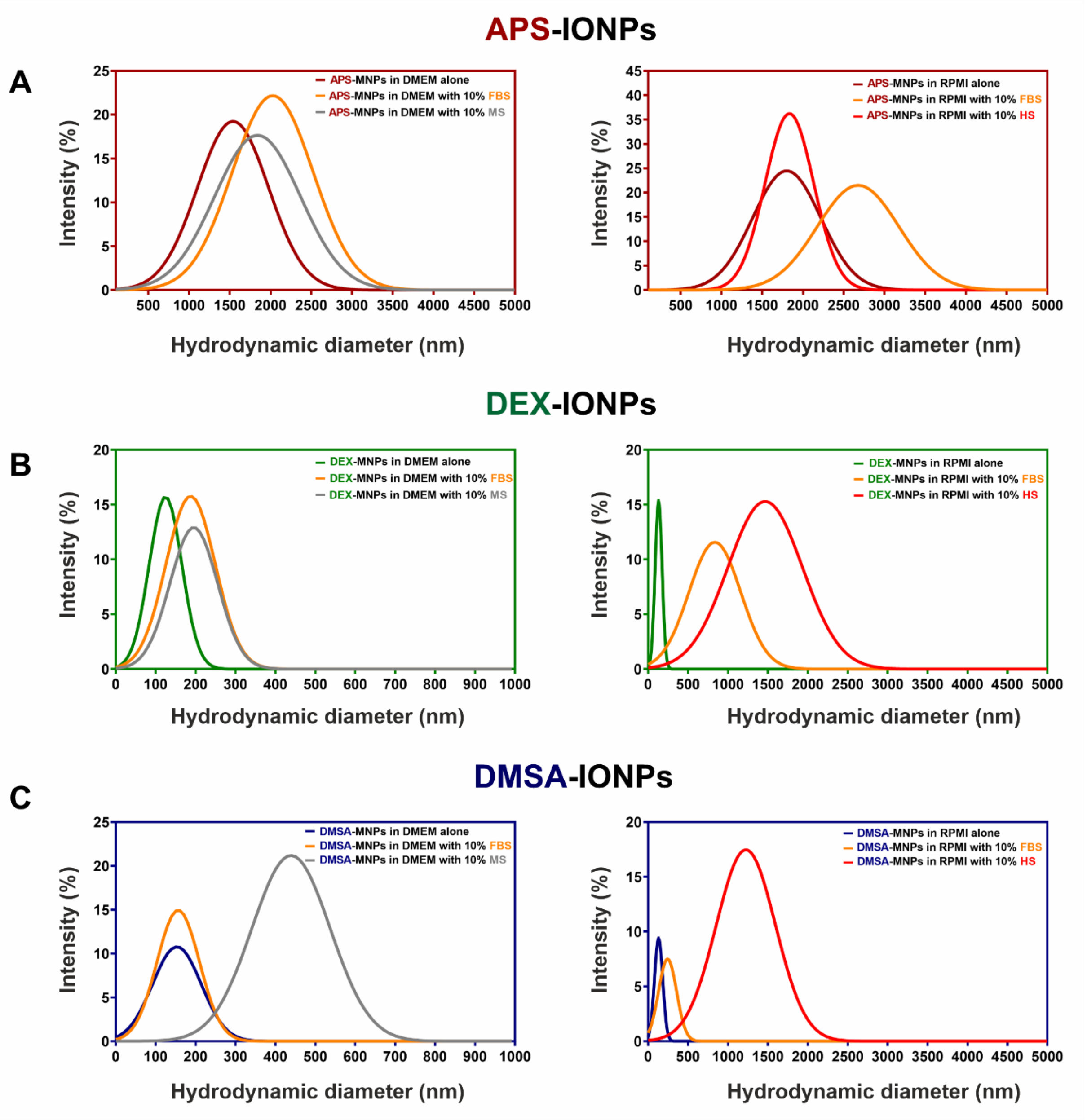

Figure S3. Characterization of the stability of IONPs at 24h. The hydrodynamic size of the (A) APS-IONPs, (B) DEX-IONPs and (C) DMSA-IONPs at 24h determined by DLS. Control sample: IONPs incubated in medium alone. Corona sample: IONPs incubated in medium (DMEM or RPMI) supplemented with different biological sera.

Characterization of the PC on the IONP surface based on the species origin of the serum. The proteins identified in the three PCs are shown in Figure S3 as a function of the origin of the biological serum and analyzed with the Panther Gene List software that recognizes Gene Ontology annotations. The analysis centered on molecular function and the type of proteins. No significant differences in function were observed between the three types of IONPs, whereas differences were observed for the same IONP when incubated in the different biological sera (see **Figure S4A**). In the case of APS-IONPs, the main differences were observed between FBS and the sera that simulate *in vivo* conditions (MS and HS), since the PC of APS-IONPs formed

in HS has distinct functional classifications relative to that formed in FBS. The main differences in the DEX-IONP PC were observed through the appearance of two new categories when the PC formed in MS and HS, in particular structural activity in the PC derived from MS and transporter activity in the PC formed in HS. Finally, no differences are observed between the main proteins categories for DMSA-IONPs: catalytic activity, binding and molecular regulatory activity. However, additional proteins involved in transporter and ATP-dependent activity were identified in the HS-derived PC.

In terms of the classes of proteins identified, three in particular were selected: apolipoproteins, complement proteins and immunoglobulins. Again, focusing on these confirmed that the greatest differences in the PC are marked by the biological serum and not the IONP coating (**Figure S4B**).

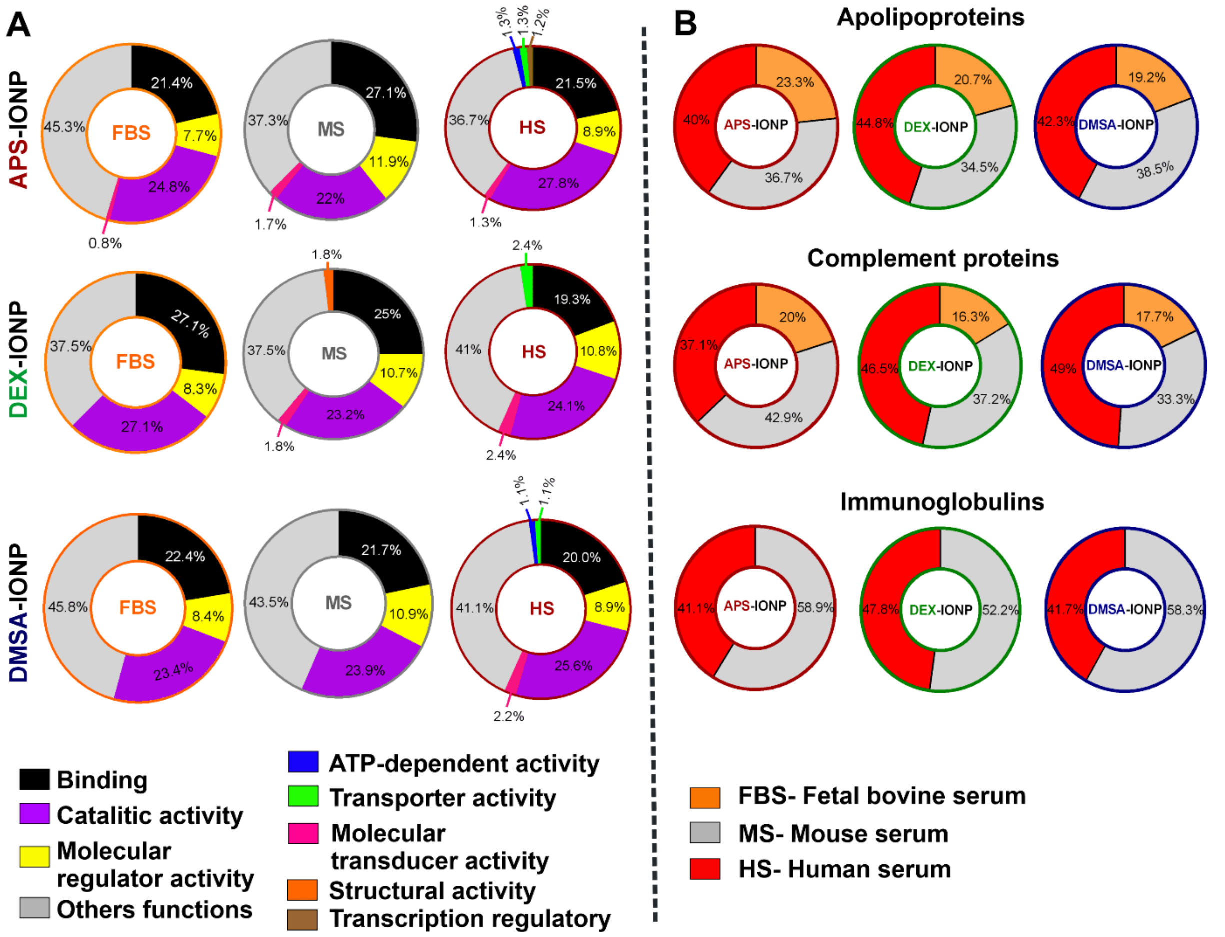

Figure S4. Comparison of the protein classes in the PCs generated in the different sera. (A) Diagrams showing the percentage of the PC of each IONP type that belongs to a given molecular function category. (B) Diagrams showing the percentage of each PC derived from each type of serum on the distinct IONPs that belong to a given class of protein. The diagrams were obtained using the Panther Gene list software.

IONP toxicity. The IONP treatment and type of sera did not affect RAW 264.7 and THP1 cell viability in PrestoBlue assays (**Figure S5**) and hence, the optimal concentration selected was 125 μg/ml for both cell types.

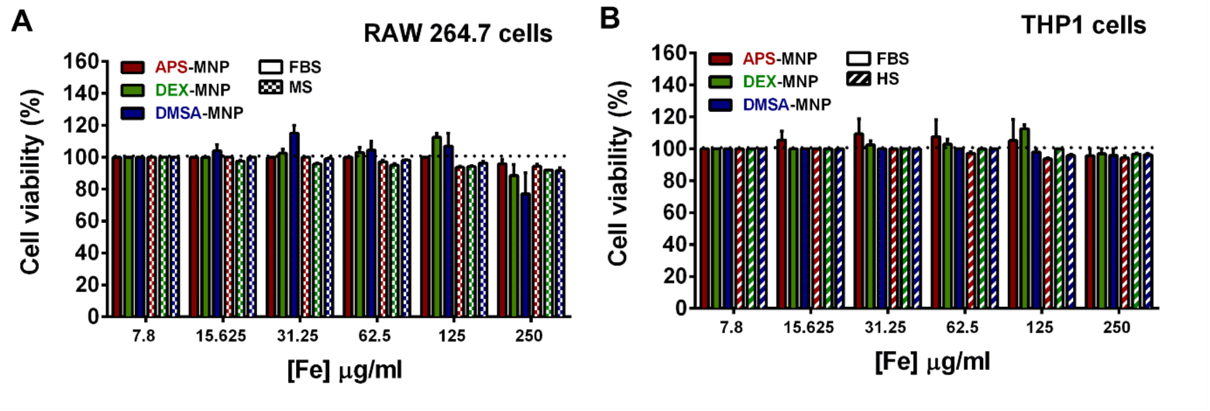

Figure S5. Evaluation of APS-, DEX- and DMSA-IONP toxicity in macrophage cells incubated in different types of sera. (A) Viability of RAW 264.7 cells after exposure to the different IONPs measured with the PrestoBlue fluorometric test. (B) Viability of THP1 cells after exposure to the different IONPs measured with the PrestoBlue fluorometric test. The data (mean ± SD) are representative of two independent experiments.

IONP uptake by macrophage cells in different biological sera. We characterized the APS-, DEX- and DMSA-IONP uptake by macrophage cells and we found RAW264.7 cells took up more APS-IONP (69.1 ±15.9 pg/cell) than DMSA-IONPs (39.0 ±5.4 pg/cell) and DEX-IONPs (16.0±5.5 pg/cell) when incubated in 10 % FBS (**Figure S6A**). Similarly, the RAW264.7 cells internalized more APS-IONPs (110.3 ±4.5 pg/cell) when incubated in 10 % MS. However, DEX-IONPs (42.0 ±12.3 pg/cell) and DMSA-IONPs (61.9 ±8.8 pg/cell) were internalized similarly by these cells when incubated in 10 % MS (**Figure S6A**). APS-IONPs also showed the highest internalization rate by THP1 cells after 24 h in either FBS (73.4 ±3.3 pg/cell) or HS (204.5 ±56.2 pg/cell), while DEX-IONPs (FBS 67.9 ±9.1 pg/cell, HS 22.5 ±12.1 pg/cell) and DMSA-IONPs (FBS 2.4 ±1.0 pg/cell, HS 1.7 ±1.0 pg/cell) showed no significant difference in cell internalization regardless of the serum (**Figure S6B**).

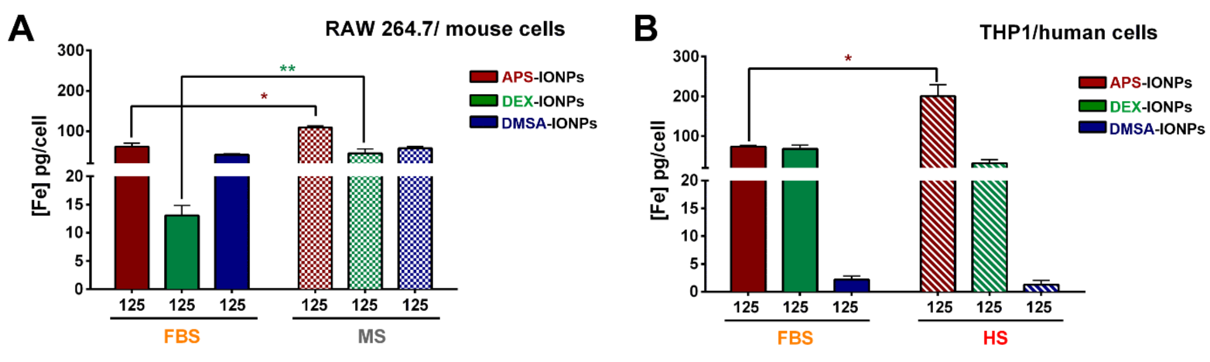

Figure S6. Cellular iron concentrations in macrophage cells after APS-, DEX- and DMSA-IONP uptake. (A) Quantification of the iron concentration in RAW 264.7 cells by ICP-OES. (B) Quantification of the iron concentration in THP1 cells by ICP-OES. The data (mean ± SD) are representative of three independent experiments. One-way analysis of variance (ANOVA) and a Student's t-test were used to assess the ICP-OES data, and the asterisks indicate significant differences: *p < 0.05 and **p < 0.01. *Legend:* FBS (fetal bovine serum), MS (mouse serum) and HS (human serum).

To verify the uptake of the IONPs with different coatings observed by ICP-OES in macrophage cells, Prussian Blue iron staining with and neutral red counterstaining was performed. After a 24 h incubation with the IONPs in the different sera, we observed the internalization differed depending on the IONP coating and the type of biological serum (see **Figure S7**).

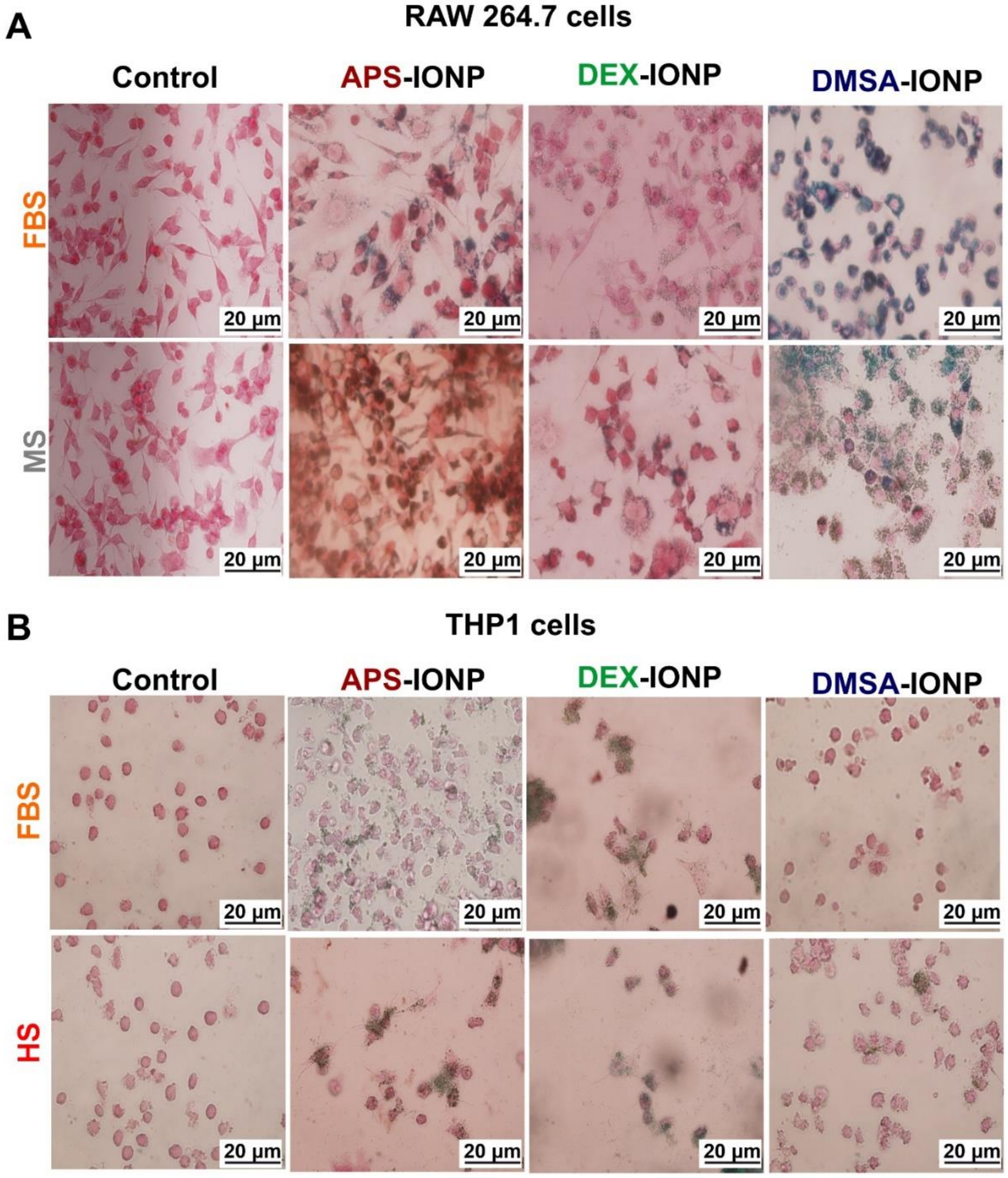

Figure S7. APS-, DEX- and DMSA-IONPs uptake by macrophage cells after a 24 h exposure in medium supplemented with different sera. (A) Perl's Prussian Blue staining of RAW 265.7 cells after exposure to IONPs. (B) Perl's Prussian Blue staining of THP1 cells after exposure to IONPs. Scale bar: 20 µm.

Influence of the biological identity of IONPs on macrophage phenotype. Below are the histograms of the cytometric analysis performed for the CD80 and CD86 markers in the RAW264.7 and THP1 macrophage cells incubated in the different types of biological sera (see **Figure S8**).

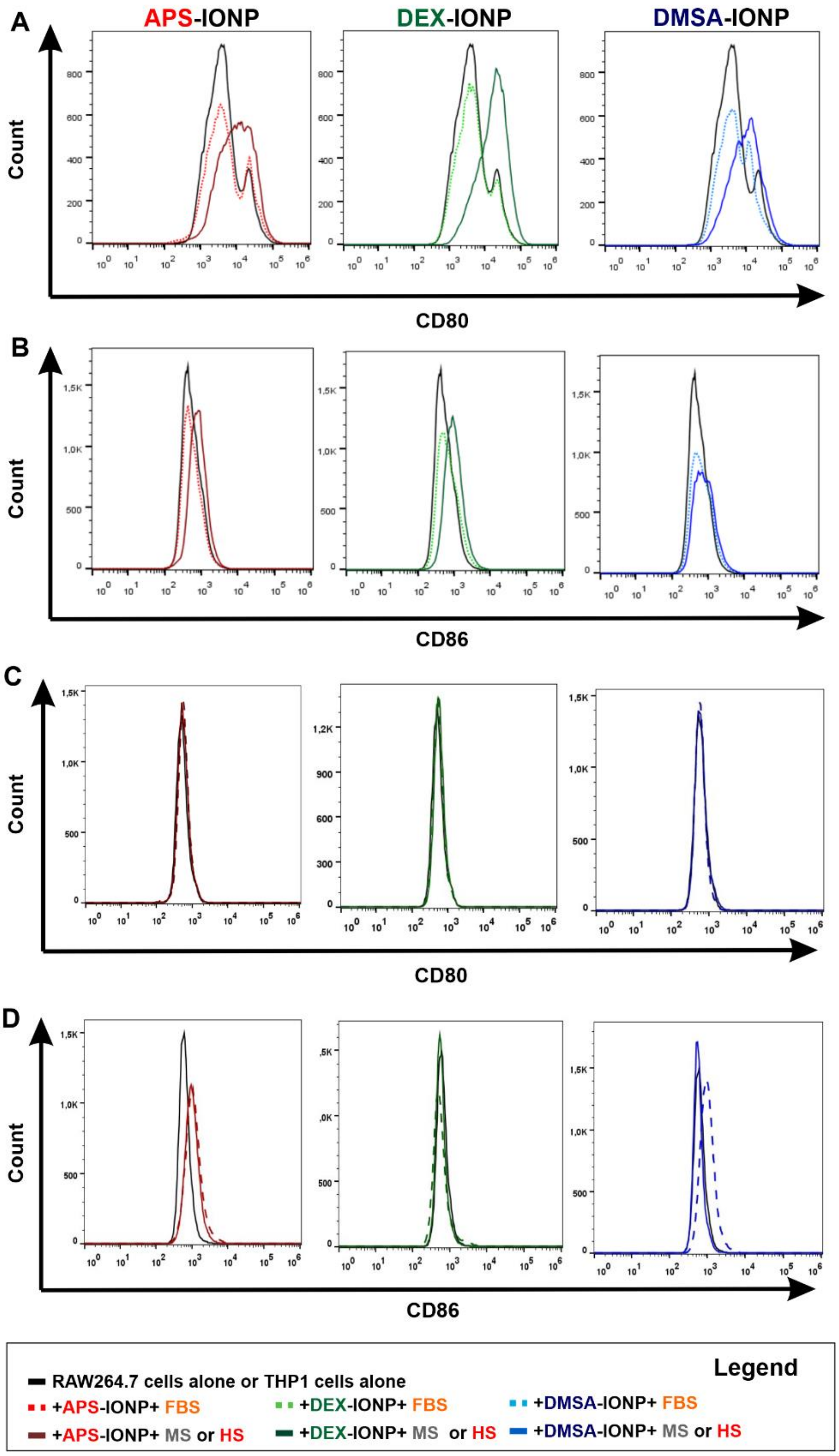

Figure S8. The influence of the PC on macrophage activation. (A, B) Histograms showing the expression of CD80 and CD86 in RAW264.7 cells. (C, D) Histograms showing the expression of CD80 and CD86 in THP1 cells. The RAW 264.7 cells were incubated in medium supplemented with 10% FBS

or 10% MS, while the THP1 cells were incubated in medium with 10% FBS or 10% HS. The histograms are representative of five independent experiments in all cases.

In summary, for RAW264.7 cells we observed differences in the influence of PC derived from FBS and MS sera for both markers used (**Figure S8A, B**), however, in THP1 cells no differences were shown in the expression of CD80 and CD86 derived from the influence of PC formation in different biological sera (**Figure S8C, D**).

Influence of the type of biological serum on macrophage migration. The differences in RAW macrophage cell migration were analyzed depending on the type of biological serum with which the culture medium was supplemented. To analyze the migration of RAW cells in DMEM supplemented with 10% FBS or MS, a wound closure assay was used and migration was analyzed over 24h. The directional migration index (DMI) was obtained using the formula indicated in the Materials and Methods [2]. These results showed significant differences in the migration of RAW 264.7 cells when cultured in medium supplemented with 10% FBS or MS, with enhanced migration when cells were cultured in medium supplemented with FBS (see **Figure S9**).

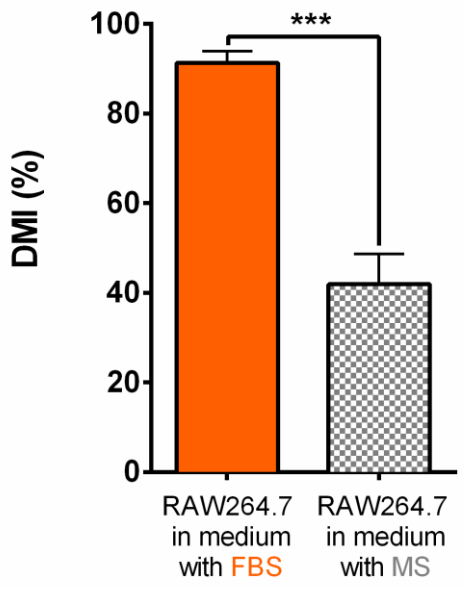

Figure S9. The directional migration index (DMI) of RAW264.7 cells cultured in medium supplemented with different sera. The DMI was measured with Image J software from three independent experiments. One-way analysis of variance (ANOVA) and a Student's t-test were used and the asterisks indicate significant differences: *$p < 0.05$, **$p < 0.01$, and ***$p < 0.001$.

Analysis of mitochondrial morphology according to the type of biological serum used after exposure to DMSA-IONPs. To analyze ROS production, the mitochondrial morphology was assessed and striking changes were observed, mainly in the RAW 264.7 murine macrophages when exposed to DMSA-IONPs in medium supplemented with FBS or MS.

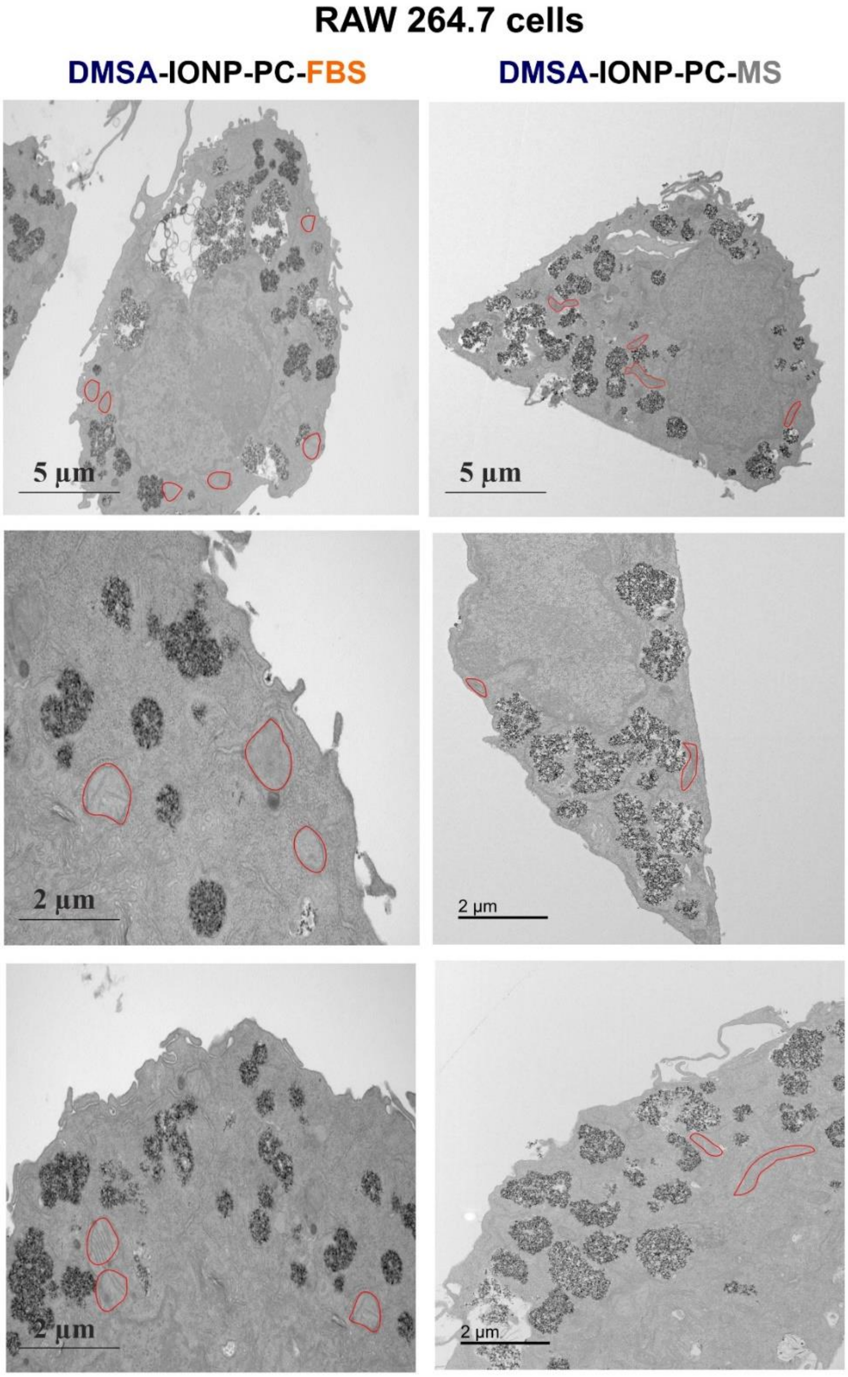

Figure S10. Study of mitochondrial morphology in RAW 264.7 cells treated with DMSA-IONPs with different PCs depending on the type of biological serum. Representative TEM images of mitochondria (in red) in RAW 264.7 cells exposed to DMSA-IONPs in medium supplemented with 10% FBS or MS. Scale bar: 2-5 µm.

When a PC was generated in FBS (DMSA-IONP-PC-FBS) the morphology of the mitochondria was spherical, while when incubated in MS (DMSA-IONP-PC-MS) they begin to change their morphology towards more elongated mitochondria. This has been related previously to the polarization of macrophages towards a M1 or M2 phenotype [3], suggesting that the PC not only changes mitochondrial metabolism but also, it influences macrophage behavior.

SUPPLEMENTARY REFERENCES

1. Portilla, Y.; Mellid, S.; Paradela, A.; Ramos-Fernández, A.; Daviu, N.; Sanz-Ortega, L.; Pérez-Yagüe, S.; Morales, M. P.; Barber, D. F., Iron Oxide Nanoparticle Coatings Dictate Cell Outcomes Despite the Influence of Protein Coronas. *ACS Applied Materials & Interfaces* **2021,** *13* (7), 7924-7944.
2. Mulens-Arias, V.; Rojas, J. M.; Pérez-Yagüe, S.; Morales Mdel, P.; Barber, D. F., Polyethylenimine-Coated SPION Exhibits Potential Intrinsic Anti-Metastatic Properties Inhibiting Migration and Invasion of Pancreatic Tumor Cells. *J Control Release* **2015,** *216*, 78-92.
3. Li, Y.; He, Y.; Miao, K.; Zheng, Y.; Deng, C.; Liu, T.-M., Imaging of Macrophage Mitochondria Dynamics In Vivo Reveals Cellular Activation Phenotype for Diagnosis. *Theranostics* **2020,** *10* (7), 2897-2917.
